# Occurrence of Lymphangiogenesis in Peripheral Nerve Autografts Contrasts Schwann Cell-Induced Apoptosis of Lymphatic Endothelial Cells In Vitro

**DOI:** 10.3390/biom12060820

**Published:** 2022-06-12

**Authors:** Carina Hromada, Jaana Hartmann, Johannes Oesterreicher, Anton Stoiber, Anna Daerr, Barbara Schädl, Eleni Priglinger, Andreas H. Teuschl-Woller, Wolfgang Holnthoner, Johannes Heinzel, David Hercher

**Affiliations:** 1 Department Life Science Engineering, University of Applied Sciences Technikum Wien, 1200 Vienna, Austria; carina.hromada@technikum-wien.at (C.H.); ansdcso@gmail.com (A.D.); andreas.teuschl@technikum-wien.at (A.H.T.-W.); 2 Austrian Cluster for Tissue Regeneration, 1200 Vienna, Austria; jaana.hartmann@trauma.lbg.ac.at (J.H.); johannes.oesterreicher@trauma.lbg.ac.at (J.O.); antonstoiber@gmail.com (A.S.); barbara.schaedl@trauma.lbg.ac.at (B.S.); eleni.priglinger@trauma.lbg.ac.at (E.P.); wolfgang.holnthoner@trauma.lbg.ac.at (W.H.); 3 Ludwig Boltzmann Institute for Traumatology, The Research Centre in Cooperation with AUVA, 1200 Vienna, Austria; jheinzel@bgu-tuebingen.de; 4 University Clinic of Dentistry, Medical University of Vienna, 1090 Vienna, Austria; 5 Department of Hand-, Plastic, Reconstructive and Burn Surgery, BG Unfallklinik Tuebingen, University of Tuebingen, 72076 Tuebingen, Germany

**Keywords:** peripheral nerve regeneration, lymphangiogenesis, Schwann cells, lymphatic endothelial cells

## Abstract

Peripheral nerve injuries pose a major clinical concern world-wide, and functional recovery after segmental peripheral nerve injury is often unsatisfactory, even in cases of autografting. Although it is well established that angiogenesis plays a pivotal role during nerve regeneration, the influence of lymphangiogenesis is strongly under-investigated. In this study, we analyzed the presence of lymphatic vasculature in healthy and regenerated murine peripheral nerves, revealing that nerve autografts contained increased numbers of lymphatic vessels after segmental damage. This led us to elucidate the interaction between lymphatic endothelial cells (LECs) and Schwann cells (SCs) in vitro. We show that SC and LEC secretomes did not influence the respective other cell types’ migration and proliferation in 2D scratch assay experiments. Furthermore, we successfully created lymphatic microvascular structures in SC-embedded 3D fibrin hydrogels, in the presence of supporting cells; whereas SCs seemed to exert anti-lymphangiogenic effects when cultured with LECs alone. Here, we describe, for the first time, increased lymphangiogenesis after peripheral nerve injury and repair. Furthermore, our findings indicate a potential lymph-repellent property of SCs, thereby providing a possible explanation for the lack of lymphatic vessels in the healthy endoneurium. Our results highlight the importance of elucidating the molecular mechanisms of SC–LEC interaction.

## 1. Introduction

Peripheral nerve injuries pose a major clinical concern world-wide, affecting up to 60,000 in 1,000,000 individuals every year [1,2]. Moreover, 20 million people are estimated to be suffering from some form of neuropathy in the US alone, resulting in approximately USD 150 billion of annual health care costs for the treatment, care, and rehabilitation of patients, mainly caused by the low success rate of both, spontaneous and surgical peripheral nerve repair [1,2,3,4]. The consequences pose a major socioeconomic burden, due to the significant deterioration or even persisting disability of patients associated with loss of sensory and/or motor function and unemployability; thereby strongly deteriorating the patients’ quality of life [3,5,6]. Moreover, peripheral nerve damage often leads to neuropathic pain; a serious condition affecting 6–8% of the population and accounting for 20–25% of all chronic pain disorders [7,8,9].

The peripheral nervous system has a remarkable regenerative capacity, which can be mainly attributed to the Schwann cells’ remarkable plasticity, allowing them to adopt a regeneration-promoting repair phenotype. These Schwann cells (SCs) participate in the degradation of myelin by autophagy, attract macrophages, activate neurotrophic factor expression, and migrate to form a cellular bridge, called bands of Büngner, to guide regenerating axons [10,11]. However, if peripheral nerve damage is too severe, the intrinsic regenerative capacity is not sufficient to functionally recover the damaged nerve and surgical interventions become necessary. The current gold standard of surgical treatment options for segmental tissue loss after nerve injury are nerve autografts, but their use has certain limitations: first, the limited availability of suitable autologous nerves; and second, the occurrence of sensory and/or motor function deficits at the donor site [11,12,13]. The alternatives to decellularized allografts are, on the other hand, associated with poorer clinical outcomes [13,14,15]. This highlights the clinical relevance of peripheral nerve injuries and the urgent need for improved repair methods.

The success rate of novel therapeutic approaches to treat peripheral nerve defects might be greatly enhanced by advancing our still rather rudimental understanding of the structure and function of the peripheral nerve itself. The anatomy of a nerve is often pictured as a cable-like structure: it consists of myelinated nerve fibers—i.e., axons surrounded by SCs—that are enclosed by the endoneurium and bundled into nerve fascicles by the perineurium. The outermost layer of connective tissue, called the epineurium, in turn bundles several fascicles into the entire nerve. This can be further subdivided into the epifascicular epineurium, which surrounds the whole nerve trunk, and the interfascicular epineurium, which spans the space between the individual fascicles [16]. First described by German anatomist Johannes Lang, but still a matter of scientific debate, is the paraneurium, a layer of loose connective tissue enabling gliding of the nerve trunk [17,18]. Furthermore, peripheral nerves have a longitudinal extrinsic vascular system surrounding the nerve, which consists of larger vessels that provide blood supply for the epi- and perineurial space, and a longitudinal intrinsic microvascular system for the endoneurial space [19]. While the organization of the blood vasculature of peripheral nerves, as well as its importance during peripheral nerve regeneration has been well described [20,21,22], studies investigating the anatomy and physiology of the neural lymphatic system are scarce [23]. Prospero Homeobox 1 (Prox-1) has been identified as the key molecule for lymphangiogenesis and nerve regeneration following crush injury of the murine sciatic nerve [24]. It has also been shown that the formation of new blood vessels after nerve transection guides migrating Schwann cells to bridge the defect, which in turn guide regenerating axons [21]; whereas, the role of the lymphatic system in neuropathies has remained widely unexplored, to date.

Sir Sidney Sunderland was the first to describe the existence of two distinct lymphatic capillary networks inside peripheral nerves: one in the epineurium and one in the endoneurial compartment, which are separated from each other by the perineurium [25]. However, until the early 2000s, the observation of lymphatic vasculature mainly relied on ultrastructural examinations, due to the lack of marker molecules specific for lymphatic vessels. In 2006, Volpi et al. first reported the immunohistochemical identification of lymphatics in the epineurium, but not in the endoneurium, of human sural nerves [26]. Given that peripheral nerve repair is frequently accompanied by endoneurial fibrosis, swelling, and local inflammation—all of which represents characteristics of a dysfunctional lymphatic system Frueh et al. recently hypothesized that the lymphatic system might play an essential role during peripheral nerve injury and repair [27]. Frueh et al. explicitly highlighted the immense gap of knowledge regarding the neural lymphatic system and suggest thorough histological investigations of peripheral nerve specimens and in vivo analysis of lymphangiogenesis of physiological and pathological peripheral nerves, as well as in vitro studies to elucidate the interplay between lymphatic endothelial cells and SCs or neuronal cells [27]. Recently, the same group investigated failed human sensory nerve allografts associated with lack of reinnervation and neuropathic pain, and showed that allograft failure was characterized by increased intraneural fibrosis, abnormal neurofilament organization, increased and randomly distributed blood microvasculature, and, most interestingly, scarce lymphatic vasculature compared to healthy nerves [28].

With this study, we aimed to provide fundamental insights regarding the role of the lymphatic system after peripheral nerve injury in vivo and to elucidate the interplay of lymphatic endothelial cells (LECs) and SCs in vitro. After an in vivo study of segmental nerve damage with autologous nerve grafting in a rat model, we observed an increase in lymphatic vessels in the nerve graft. This sparked our interest and led us to investigate the interaction between primary SCs and primary LECs in 2D and 3D in vitro experiments. Specifically, we investigated the influence of pro-regenerative SCs, as well as their secretome, on lymphatic vessel formation in 3D fibrin hydrogels and the migration of LECs in a 2D scratch assay, respectively. Taken together, our findings will not only shed light onto the involvement of the lymphatic system in injured peripheral nerves, but also pave the way for the establishment of novel therapeutic approaches for improved nerve regeneration.

## 2. Materials and Methods

### 2.1. Ethical Approval

The experimental protocol was approved beforehand on 23 July 2019, by the Animal Protocol Review Board of the City Government of Vienna (Magistrate’s office number 58, Project identification code: MA58-421715-2019-16). All procedures were carried out in full accordance with the Helsinki Declaration on Animal Rights and the Guide for the Care and Use of Laboratory Animals of the National Institutes of Health.2.2 Animals

Nine male Lewis rats (Janvier Labs, Le Genest-Saint-Isle, France), weighing 280–360 g, underwent bilateral surgical transection of the median nerve with unilateral repair using autologous nerve graft (ANT, *n* = 9). The animals were kept in groups of two or three in appropriate cages with ad libitum access to food and water. Following their arrival at the animal facility, rats were allowed a 7-day acclimatization period before any experimental handling.

### 2.2. Experimental Surgery

Following acclimatization, rats underwent bilateral median nerve resection, as described previously [29]. All surgeries were performed under aseptic conditions using an operation microscope (Leica M651, Leica Microsystems, Vienna, Austria). Anesthesia was maintained by an oxygen-anesthetics mixture (95–98.5% oxygen, 1.5–5% sevoflurane) through inhalation via a nose cone. For sufficient peri- and post-operative anesthesia, buprenorphine (0.05 mg/kg) was injected subcutaneously preoperatively and every 8 h postoperatively for at least 24 h. Additionally, Meloxicam (0.75 mg/kg) was applied orally for at least 4 days following surgery. After careful dissection of the surrounding tissue, 7 mm of the left and right median nerve were removed after performing a microsurgical transection about 1.5 mm proximal to the crossing point of the median nerve and brachial artery and vein. Two minutes prior to transection, two drops of 2% lidocaine were applied to the nerve. The second transection was performed 7 mm proximal to the first one. In the right forelimb, the nerve gap was bridged with the original nerve segment in reverse fashion, serving as a homotopic nerve autograft, which was coaptated to the nerve stumps with two interrupted sutures per coaptation site (Ethilon R 10-0, Ethicon-Johnson & Johnson, Brussels, Belgium). In the left forelimb, the nerve defect remained unreconstructed and the distal nerve stump was sutured into the short head of the bicep muscle to prevent reinnervation. The subcutaneous tissue and skin were closed with interrupted absorbable sutures (Vicryl 5-0, Johnson & Johnson, Vienna, Austria).

### 2.3. Tissue Preparation for Histological Analysis

At the end of the 12-week postoperative observational period, animals were sacrificed in deep anesthesia, as described before, by means of an intracardially applied overdose of sodium thiopental. Immediately following sacrification, a segment of the right median nerve was harvested containing the reconstructed nerve segment, as well as 5 mm of the original nerve proximal and distal to it.

To obtain the correct position, as well as distal and proximal orientation of the nerves, they were pinned with minutien needles on small Styrofoam stubs. For the histochemical and immunohistochemical stainings, the nerves were fixed in 4% buffered formalin for 24 h at room temperature and afterwards rinsed in tap water for 1 h. Dehydration with an uprising ethanol series was performed, beginning with 50% EtOH for 1 h, followed by 70% EtOH. Then, the samples were transferred to a vacuum infiltration processor (Sakura, TissueTek^®^ VIP, Sakura Finetek Germany GmbH, Umkirch, Germany) and after further dehydration of the samples, infiltrated with paraffin via the intermedium of xylene. Cutting the nerve samples into 4-µm thin cross-sections was performed with a Microm HM355S (Thermo Scientific, Waltham, MA, USA). After drying the sections overnight in a 37 °C oven, the slides were deparaffinized and rehydrated for staining with different methods. Nuclei were stained in grey using Weigert’s Iron Heamatoxylin. After staining, the sections were dehydrated and permanently embedded with Shandon Consul-Mount (Thermo Scientific). Starting immunohistochemical stainings, the sections for podoplanin (ReliaTech, Wolfenbüttel, Germany, 104-M40) staining were steamed in a pH 6 sodium citrate buffer (0.1 M) for 20 min, for antigen retrieval. After the antigen retrieval, the sections were blocked using Bloxall^®^ (VectorLabs, Newark, CA, USA) for 10 min. Then, the primary antibody podoplanin (1:2000) was applied for 1 h at room temperature, followed by incubation of secondary antibody for 30 min at room temperature using an HRP conjugated anti-mouse system (ImmunoLogic, VWRKDPVM110HRP). The detection of the staining was performed with ImmPACTTM NovaREDTM (VectorLabs). Then the sections were counterstained with Haematoxylin and after dehydration permanently embedded with Shandon Consul-Mount (Thermo Scientific).

### 2.4. Automated Quantification of Lymphatic Vessels

Automated, deep learning-based image analysis was used to evaluate lymphatic vessels in whole-slide scans of histological cross sections. We adopted the IKOSA platform (KML Vision, Graz, Austria) to train a state-of-the-art deep convolutional neural network (CNN) model to segment and measure the podoplanin-stained vessels in the scans. The trained application provides a set of morphometric parameters that are calculated automatically for each detected object. To improve the quality of data annotations for supervised learning, we used regions of interest (ROI) to restrict the image content and marked the vessels with the annotation tools provided by IKOSA. Similarly, we used ROI to define areas without lymphatic vessels, to aid the model in distinguishing between foreground and background. A set of 13 scans was randomly split into training (10 images, 233 ROI) and validation (3 images, 42 ROI). Training was performed on a GPU-accelerated cloud infrastructure, the semantic segmentation performance was evaluated on the validation set using Dice coefficient, precision, and recall metrics, see Supplementary File S1 for more details.

### 2.5. Cell Culture

SCs were dissected from collected sciatic nerves from adult female and male Lewis rats, according to a protocol modified from Kaekhaw et al. [30] and Weiss et al. [31]. Briefly, the epineurium was removed from sciatic nerves and nerve fascicles were enzymatically digested in Dulbecco’s Modified Eagle’s Medium High Glucose (DMEM HG, Lonza, Basel, Switzerland) containing 0.1% Collagenase Type I ((*w*/*v*), Sigma Aldrich, St. Louis, MO, USA), 1.25% Dispase I ((*w*/*v*), Sigma Aldrich, St. Louis, MO, USA), 3 mM CaCl_2_, 1% Penicillin/Streptomycin (P/S, Lonza) and 1% Amphotericin B (Lonza) at 37 °C and 5% CO_2_ overnight. The next day, digested fascicles were triturated, the suspension was filtered through a 70 µm nylon cell strainer, and DMEM HG (Lonza) supplemented with 10% fetal calf serum (FCS, GE Healthcare, Chicago, IL, USA) was added. After centrifugation at 300 *g* for 5 min, the cell pellet was resuspended in Schwann cell medium consisting of DMEM HG D-Valine (HiMedia Laboratories GmbH, Einhausen, Germany) supplemented with 10% FCS, 1% P/S, 1% L-Glutamine, 10 ng/mL heregulinβ-1 (PeproTech, #100-03) and 2 µM forskolin (Sigma Aldrich, St. Louis, MO, USA), and seeded onto 0.01% poly-L-lysine (Sigma Aldrich, St. Louis, MO, USA) and 1 µg/mL laminin (Sigma Aldrich, St. Louis, MO, USA) coated plates. To eliminate fibroblasts, medium supplemented with the non-essential amino acid D-Valine instead of the essential L-valine amino acid was used. In contrast to SCs, fibroblasts do not express the enzyme D-amino acid oxidase (DAAO) and, thus, cannot metabolize D-valine into the essential L-valine, resulting in fibroblast death, due to the lack of the essential L-valine amino acid [30]. To further purify SCs from any remaining fibroblasts, the differential adhesion properties of SCs and fibroblasts were exploited [31]. Briefly, SCs were detached with ice cold Trypsin-Versene (Lonza) for 2 min at room temperature, centrifuged at 300 *g* for 5 min, and the cell suspension was seeded onto uncoated 6-wells. After incubation at 37 °C, 5% CO_2_ for 30 min, mainly fibroblasts attached to the culture surface and the supernatant containing mostly SCs were centrifuged at 300 *g* for 5 min and cells subsequently seeded onto poly-L-lysine/laminin-coated 6-wells. With this combined approach, cultures with a high purity of >97% could be achieved (Supplementary Figure S1). Medium was exchanged every two to three days and cells were subcultivated at a confluency of approximately 90%.

LECs were isolated from the dermal microvascular endothelial cell population of human juvenile foreskin, which was obtained with informed consent and ethical approval (Ethic Committee Charité University Medicine, Berlin, Germany) after routine circumcisions [32]. As previously described [33], LEC were isolated by immunomagnetic cell sorting using anti-podoplanin antibody and magnetic beads (Dynabeads, M280 goat anti-rabbit, Thermo Fisher) according to the manufacturer’s instructions. The podoplanin-positive LEC were maintained in Endothelial Cell Growth Medium-2 (EGM-2) consisting of Endothelial Cell Basal Medium-2 containing 5% FCS and endothelial growth supplements (EGM-2 Bulletkit, Lonza), as well as 25 ng/mL VEGF-C (ReliaTech, #300-079). LEC were retrovirally infected with enhanced yellow fluorescent protein (eYFP) to visualize the vascular network formation in 3D fibrin hydrogels, as described previously [33].

Human adipose-derived stem/stromal cells (ASCs) were isolated from liposuction material, as described previously [34], with prior approval by the ethics committee of Upper Austria (ethics vote #200), and maintained in EGM-2.

#### Characterization of Schwann Cell Purity

For SC characterization via immunofluorescence, 20,000 SCs were seeded onto poly-L-lysine coated 12-mm round glass coverslips (VWR). Cells were fixed in 4% formaldehyde (ROTI Histofix, Carl Roth, Karlsruhe, Germany) for 20 min at room temperature, followed by three washing steps in Dulbecco’s phosphate buffered saline (DPBS, Lonza). For staining of the cell surface receptor NGFR, cells were blocked with DPBS containing 1% bovine serum albumin (BSA, Sigma Aldrich, St. Louis, MO, USA) and 3% goat serum (Biowest, Nuaillé, France) for 30 min at room temperature. For staining of the intracellular proteins SOX10 and cJun, cells were blocked and permeabilized in DPBS containing 1% bovine BSA, 3% goat serum, and 0.3% Triton X-100 for 20 min at room temperature. Subsequently, primary antibody incubation was performed in DPBS with 1% BSA, 3% goat serum for mouse monoclonal NGFR (1:50, Santa Cruz Biotechnology, Dallas, TX, USA), or in DPBS containing 1% BSA, 3% goat serum, and 0.1% Triton X-100 for mouse monoclonal cJun and mouse monoclonal SOX10 (both 1:50, Santa Cruz Biotechnology) at 4 °C overnight. After three washing steps in DPBS, cells were incubated in goat anti-mouse AlexaFluor 488 secondary antibody (1:400, ThermoFisher Scientific, Waltham, MA, USA) at 37 °C for 1 h. After another three washes in DPBS, nuclei were counterstained with DAPI (1:1000), three washing steps in DPBS were performed and coverslips were mounted with Fluoroshield (Sigma Aldrich, St. Louis, MO, USA) on glass slides. Images were taken using a Leica THUNDER Imager Live Cell & 3D Assay microscope.

To also analyze SC purity quantitatively, flow cytometry analysis using the glial marker S100 was performed. Briefly, 1 × 10^6^ cells were fixed in 1% formaldehyde for 15 min on ice. After centrifugation at 300× *g* for 5 min, cells were permeabilized via the dropwise addition of 90% ice cold methanol under constant vortexing at low speed and incubated for 30 min on ice. Subsequently, cells were washed three times in DPBS containing 1% BSA (PBS/BSA) and stained with rabbit polyclonal anti-S100 antibody (1:400, DAKO Agilent, Santa Clara, CA, USA, Z0311) in PBS/BSA for 30 min on ice. After three washing steps in PBS/BSA, cells were incubated in goat anti-rabbit AlexaFluor 488 secondary antibody (1:400, ThermoFisher Scientific, Waltham, MA, USA) for 30 min on ice in the dark. After the final washing step, cells were resuspended in 300 µL PBS/BSA and analyzed on a BD FACS Canto II (BD Biosciences, Franklyn Lakes, NJ, USA) flow cytometer. Cells stained with only the secondary antibody served as controls. Data were analyzed using FlowJo software version 10.4.2 (Tree Star, Ashland, Wilmington, DE, USA).

### 2.6. 2D Scratch Assay

Schwann cells or LEC were seeded onto 6-wells and grown until confluency in Schwann cell medium or EGM-2, respectively. Then, 24 h before the experiment, media were exchanged for a 1:1 mixture of Schwann cell medium and EGM-2. The supernatant was then collected and centrifuged at 300 *g* for 5 min. In the meantime, the monolayers were scratched with a 1000 µL pipet tip along a ruler to create a gap. After a washing step with 1x DPBS, to remove floating cells from scratching, SCs and LECs were cultivated in either SC-conditioned medium, LEC-conditioned medium, or unconditioned medium for 72 h. Images were taken at 0, 6, 20, 24, 48, and 72 h, and closure of the cell-free area was quantified with the “Wound Healing Size Tool” plugin for ImageJ/Fiji^®^ [35]. Three scratch assay experiments with different biological donors were performed, with a total of 15 technical replicates for the SC and LEC secretome groups; two scratch assay experiments with different biological donors with a total of 12 technical replicates were performed for the unconditioned media group.

### 2.7. 2D Co-Cultures

Two-dimensional co-cultures were prepared in 24-well plates, with 20,000 cells per cell type, in duplicates of the following groups: (i) YFP-LEC + ASC + SC, (ii) YFP-LEC + SC, and (iii) YFP-LEC + ASC. Cultures were maintained in a 1:1 mixture of Schwann cell medium and EGM-2 supplemented with 25 ng/mL VEGF-C for 7 days, and media was exchanged every 2–3 days. Images were acquired with a Leica DMI6000B epifluorescence microscope.

### 2.8. Cultivation of Cells in 3D Fibrin Hydrogels

Fibrin hydrogels containing different compositions of SCs, YFP-LECs, and ASCs were prepared as previously described [33]. Briefly, 100,000 YFP-LECs were embedded either (i) alone, together (ii) with 100,000 SCs and 100,000 ASCs (LEC + ASC + SC), (iii) with 100,000 SCs only (LEC + SC), or (iv) with 100,000 ASCs only (LEC + ASC) in 200 µL fibrin hydrogels on 15-mm round glass coverslips, with a final concentration of 2.5 mg/mL fibrin and 0.2 U/mL thrombin (Tissucol Duo 500 5.0 mL Fibrin Sealant, Baxter, West Deerfield Township, IL, USA). After polymerization at 37 °C and 5% CO_2_ for 15 min, a 1:1 mixture of Schwann cell medium and EGM-2, supplemented with 25 ng/mL VEGF-C and 100 U/mL aprotinin was added to fibrin hydrogels. Each hydrogel–cell composition was prepared in quadruplicate, of which 2 hydrogels each were stimulated with 10 ng/mL TNF-α for the first 48 h. Media exchanges were performed every 2–3 days. Four independent experiments were performed with three different biological donors for SCs, and two different biological donors for both, YFP-LECs and ASCs. SCs were used between passage 4 and 7, whereas YFP-LECs and ASCs were used between passages 6 and 9.

### 2.9. Immunostaining of 3D Fibrin Hydrogels

3D fibrin hydrogels were fixed on day 7 in 4% formaldehyde for 20 min at room temperature. After three washing steps in DPBS, hydrogels were blocked and permeabilized in DPBS containing 1% BSA, 3% goat serum, and 0.1% Triton X-100 for 30 min at room temperature. To stain Schwann cells, hydrogels were incubated in rabbit polyclonal anti-S100 antibody (1:400, DAKO Agilent, Z0311) in DPBS with 1% BSA, 3% goat serum, and 0.1% Triton X-100 at 4 °C overnight. After three washing steps in DPBS, hydrogels were incubated in goat anti-rabbit AlexaFluor 594 secondary antibody (1:400, ThermoFisher Scientific, Waltham, MA, USA) in DPBS with 1% BSA, 3% goat serum, and 0.1% Triton X-100 at 37 °C for 1 h. After another three washes in DPBS, nuclei were counterstained with DAPI (1:1000), and hydrogels were again washed three times in DPBS and mounted with Fluoroshield on glass slides. Images were taken at three representative areas of each fibrin hydrogel using a Leica THUNDER Imager Live Cell & 3D Assay microscope. Selected samples were scanned with a TissueFAXS tissue cytometer (TissueFAXS version 7.1.6245.112, TissueGnostics GmbH, Vienna, Austria).

### 2.10. Vascular Network Quantification

Lymphatic vascular networks were quantified using the open source tool Angiotool [36]. Main readout parameters were percentage of network vs total area, total tubule length, and number of junctions. A detailed statistics report of Angiotool network quantification is provided in Supplementary Table S1.

### 2.11. Timelapse Imaging of 2D Co-Cultures

#### 2.11.1. Nanolive Imaging

SCs were first stained with Vybrant DiD Cell-Labeling Solution (ThermoFisher Scientific, Waltham, MA, USA). Following incubation for 20 min at 37 °C and 5% CO_2_, the labeling solution was removed from the cells through centrifugation at 300 *g* for 5 min and subsequently washed once with DPBS. YFP-LECs and the DiD-labeled SCs were seeded 100,000 cells per cell type to one µ-Dish 35 mm, low (ibidi Gmbh, Gräfeling, Germany) in a total volume of 1 mL. The cells were maintained in a 1:1 mixture of Schwann cell medium and EGM-2, supplemented with 25 ng/mL VEGF-C. Before imaging could commence, the cells were incubated for 1.5 h at 37 °C and 5% CO_2_, in order to allow them to settle and adhere. After incubation, imaging of the sample with the 3D Cell Explorer (Nanolive SA, Tolochenaz, Switzerland) began. The sample was observed for a duration of 48 h. A brightfield image was acquired every 6 frames, while a fluorescence image was collected every 7 frames. YFP-LECs and SCs were used at passage 6 and 8, respectively.

#### 2.11.2. JuLI Imaging

SCs and YFP-LECs were seeded 10,000 cells per cell type to one well of a 48-well plate (ThermoFisher Scientific, Waltham, MA, USA) in a total volume of 500 µL. The cells were maintained in a 1:1 mixture of Schwann cell medium and EGM-2, supplemented with 25 ng/mL VEGF-C. After seeding, the co-culture sample was incubated at 37 °C and 5% CO_2_ for 2 h, in order to be allowed to settle and adhere. Over the span of 48 h, images were collected every 30 min in brightfield, fluorescence, and merged versions with a JuLI Smart fluorescent cell analyzer (NanoEnTek Inc, Seol, Korea). YFP-LECs and SCs were used between passage 6 and 9.

### 2.12. Statistical Analysis

Statistical analyses were performed with GraphPad Prism 9, and data are presented as mean ± standard deviation. Unless indicated differently, all data were evaluated using one-way ANOVA with Tukey post-tests to perform multiple comparisons.

## 3. Results

### 3.1. Autologous Nerve Grafts Show Increased Numbers of Lymphatic Vessels

**Figure 1:**
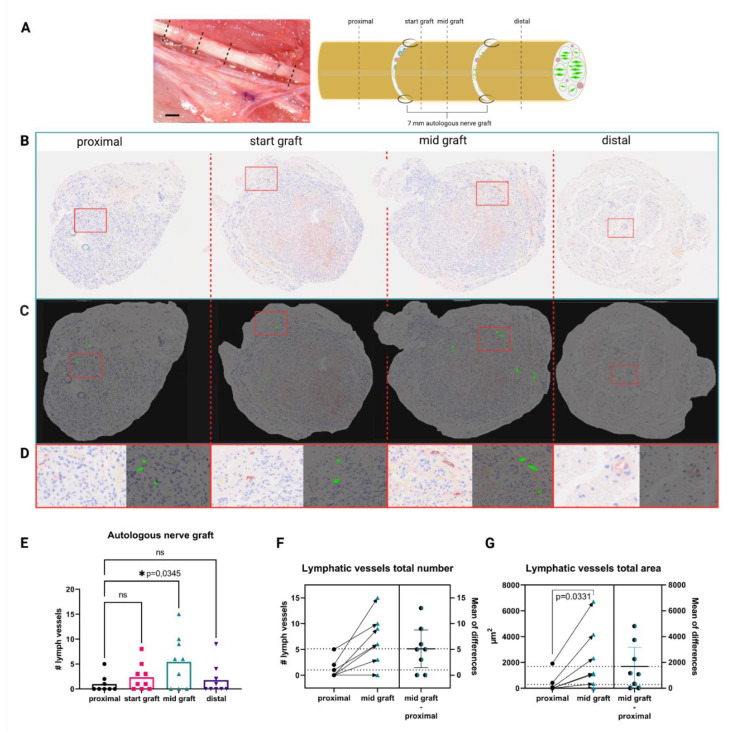
Autologous nerve grafting results in increased intraneural lymphangiogenesis. (**A**) in vivo appearance of the median nerve after autologous nerve grafting and schematic of the repaired median nerve, with histological sections and their respective localization indicated as proximal, start graft, mid graft, and distal. Dotted lines indicate histological cutting planes. Scale bar = 1 mm (**B**) Representative histological cross sections of the rat median nerve, 3 months after autologous nerve grafting stained for podoplanin. From left to right: proximal to the autologous nerve graft, start of the graft, mid-section of the graft, and distal to the graft; red rectangles indicate enlarged regions of interest depicted in (**D**). (**C**) Readout of the automated identification using KML Vision IKOSA deep learning algorithm, lymphatic vessels are highlighted in green, red rectangles indicate enlarged regions of interest depicted in (**D**). (**D**) Side-by-side comparison of respective regions of interest within histological pictures with automatically identified lymphatic vessels. (**E**) Quantification of podoplanin+ lymphatic vessels in the histological sections shows a significantly higher number of lymphatic vessels at mid graft level, when compared to the proximal nerve section. (**F**) Comparison of lymphatic vessel number proximal to mid graft in individual nerves shows increases in lymphatic vessel number in almost all samples. The average increase in vessel number from proximal to mid graft was 5; lower dotted line indicates the mean lymphatic vessel number in the proximal segment; whereas, the upper dotted line indicates the mean lymphatic vessel number in the mid graft segment. Arrows indicate changes in nerve segments of the same samples (*n* = 8). (**G**) Similar to (**F**), the comparison of the total vessel area also revealed a significant increase from the proximal to the mid graft segment of individual nerves, with an average increase of 1800 µm^2^, dotted lines represent average proximal and mid graft lymphatic vessel area (*n* = 8). (ns—not significant, * *p* < 0.05 was considered significant, asterisk indicates significance, matched one-way ANOVA with Dunnet’s test for multiple comparison was used to compare lymphatic vessel numbers in different nerve segments to the proximal nerve segment, a paired t-test was performed to compare proximal and mid graft lymphatic vessel total area).

In order to investigate the role of the lymphatic system in pathophysiological settings after segmental nerve damage, we screened for the presence of lymphatic vessels in injured median nerves at different segments of the regenerated nerve, as well as healthy uninjured median nerves. Immunohistochemical analysis revealed a significantly increased number of podoplanin-positive vessels in the mid-graft section of the autologous nerve transplant, when compared to the proximal section (Figure 1E). Analysis of individual nerves revealed significant increases of the number of lymphatic vessels (Figure 1F), as well as of the total vessel area at proximal to mid graft in almost all samples (Figure 1G). In contrast, in healthy median nerves no lymphatic vessels were found (data not shown), indicating a potential role of lymphatics in peripheral nerve regeneration, but not homeostasis. Training the CNN model took about 12 min, resulting in an overall dice score of 0.86, precision of 91.54%, and recall of 80.74% on the validation dataset. The model is robust and does not confuse blood vessels with lymphatic vessels. For a detailed performance report of the automated evaluation for each individual ROI please refer to Supplementary File S1.

### 3.2. Secretomes of Schwann Cells and Lymphatic Endothelial Cells Do Not Influence Each Others Capacity to Close Cell Monolayer Gaps

**Figure 2:**
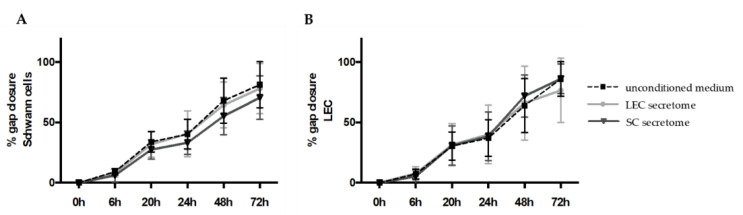
Schwann cell migration was not altered by lymphatic endothelial cell secretome in 2D scratch assay experiments. SCs and LECs were grown until confluency and their conditioned media were collected 24 h before the experiment. Scratches were created on cell monolayers and cell migration in either unconditioned medium or conditioned medium from LECs or SCs was monitored over a total of 72 h. (**A**,**B**) Quantification of gap closure showed that the secretome of LECs and SCs did not affect the migration of either SCs or LECs. Data are presented as mean ± sd. LEC secretome and SC secretome: *n* = 15 of 3 independent experiments; unconditioned medium: *n* = 12 of 2 independent experiments.

Since we observed an increased number of lymphatic vessels in autologous nerve grafts after segmental nerve damage, we aimed to investigate the interaction between primary SCs and LECs in in vitro settings. In a first step, we performed in vitro scratch assay experiments, to analyze the effect of SC and LEC secretomes on cell migration and proliferation. Therefore, confluent LEC and SC monolayers were scratched with a 1000-µL pipet tip to create a cell-free area, and scratched cell monolayers were either incubated with conditioned media derived from LECs or SCs, or with unconditioned medium, to serve as a control. Although SC migration was slightly slower with SC secretome compared to unconditioned medium or LEC secretome, this effect was not statistically significant. Similarly, there was a trend towards slower cell migration and/or proliferation of LECs when cultivated in LEC secretome compared to SC secretome or unconditioned medium, which was statistically non-significant (Figure 2A,B). Representative images of the scratch assay are provided in Supplementary Figure S2.

Overall, gap closure was achieved by both cell types, with no significant difference in cell migration and proliferation between conditioned media of either LEC or SC and unconditioned media for both cell types.

### 3.3. 2D Cultures of LEC, SC, and ASC Induce the Formation of Lymphatic Vascular Structures

**Figure 3:**
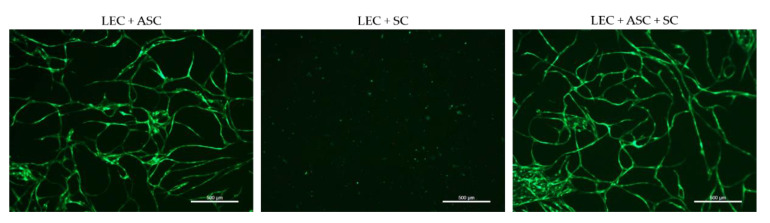
2D culture of YFP-LECs (green) with either ASCs or SCs alone, or with both ASCs and SCs. Cells were seeded in equal densities. Elongated lymphatic network-like structures formed when LECs were co-cultured with ASCs as well as with both ASCs and SCs. In contrast, LECs remained as single cells but also seemed to decrease in number when cultured with SCs alone. Green—YFP-LECs. Scale bars = 500 µm.

We next aimed to examine the influence of SCs on lymphatic tube morphogenesis in 2D cultures. Previous observations showed that LECs are able to form a vascular network when co-cultured with ASCs as a supporting cell type in 2D, as well as 3D, cultures [33]. Here, we aimed to determine whether LECs can also form networks when co-cultured either with SCs only, or with both ASCs and SCs, in 2D within seven days. Both the co-culture with ASCs and the tri-culture with ASCs and SCs resulted in the formation of elongated and interconnected vascular-like networks (Figure 3). However, LECs were not able to form tube-like structures and, moreover, appeared to decrease in number when cultured together with SCs only.

### 3.4. 3D Cultivation of LEC and SC in the Presence of ASC Induces the Formation of Lymphatic Vessel-like Structures in Fibrin Hydrogels

**Figure 4:**
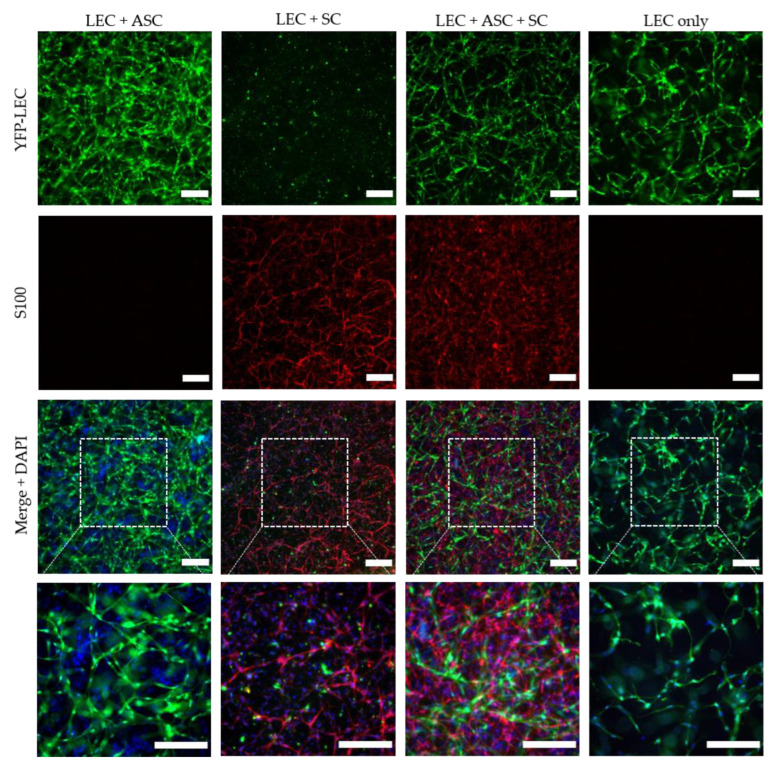
Lymphatic network formation is decreased in the presence of Schwann cells in 3D fibrin hydrogels. LECs formed elongated and interconnected networks when cultivated with ASCs as a supporting cell type (LEC + ASC). In a tri-culture of LECs, ASCs, and SCs, lymphatic vessel formation was less dense and interconnected (LEC + ASC + SC), whereas in the monoculture of LECs, only primitive tube-like structures were formed. In contrast, co-culture of only LECs and SCs inhibited the formation of lymphatic tubes. Green—YFP-LECs, red—SCs stained against S100, blue—cell nuclei. Scale bars = 200 µm.

In order to investigate the influence of SCs on lymphatic vessel formation in a more physiologically relevant setting, LECs were integrated into 3D fibrin hydrogels, either as monocultures, as co-cultures with either ASCs or SCs alone, or as tricultures with both ASCs and SCs (Figure 4). LECs formed highly dense and interconnected lymphatic networks in the presence of ASCs, which serve as a supporting cell type for vessel formation. Even though a lymphatic network also formed in tri-cultures with both ASCs and SCs, this network was less dense and interconnected compared to the co-culture with ASCs only. In accordance with the observations from the 2D experiments, LECs failed to develop vascular structures when embedded into fibrin matrices with SCs only and remained as single cells, indicating that SCs do not serve as a supporting cell type that enables tube-like formation of LECs. However, when LECs were cultivated alone, primitive elongated tube-like structures could form, whereas LECs mostly appeared round when co-cultured with SCs only, indicating that SCs might induce apoptosis on LECs.

**Figure 5:**
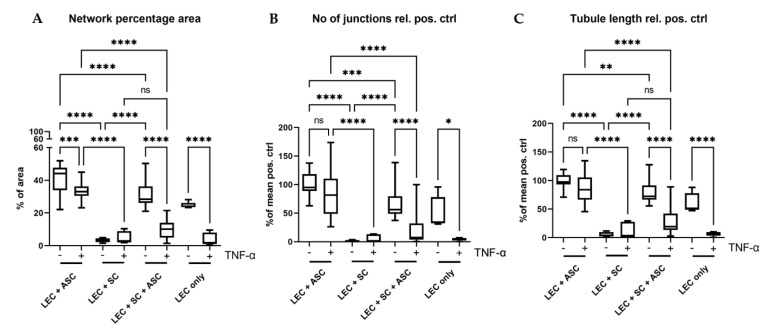
Quantification of the formed lymphatic networks in 3D fibrin clots. (**A**) Evaluation of the average area covered by lymphatic networks in percentage of total area. (**B**) Evaluation of the average number of junctions relative to the mean of the positive control (LEC + ASC). (**C**) Quantification of the average lymphatic tubule length relative to the mean of the positive control (LEC + ASC). In all parameters, the triple culture of LECs, SCs, and ASCs showed significantly lower levels than the positive control (LEC + ASC) (**A**–**C**). Co-culture of LECs with SCs inhibited network formation, whereas addition of ASCs reversed this effect. Furthermore, the effect of pro-inflammatory TNF-α on the formation of lymphatic networks was also evaluated. Here, we observed a larger negative effect of TNF-α on the triple-culture, when compared to the positive control in all parameters. LEC + ASC, LEC + SC and LEC + ASC + SC: *n* = 8 each of 4 independent experiments, LEC only: *n* = 4 of 2 independent experiments. * *p* < 0.05, ** *p* < 0.01, *** *p* < 0.001, **** *p* < 0.0001, ns not significant.

The quantification of network parameters showed a significantly larger area covered with lymphatic networks, higher numbers of junctions, as well as longer tubule length in the co-culture of LECs with ASCs compared to the tri-culture of LECs with ASCs and SCs (Figure 5). However, the higher network density in LEC and ASC co-cultures might also be attributed to the fact that a lower total cell number was embedded into the fibrin hydrogels compared to tri-culture hydrogels, thereby giving the vessel-like structures more space to spread and occupy.

**Figure 6:**
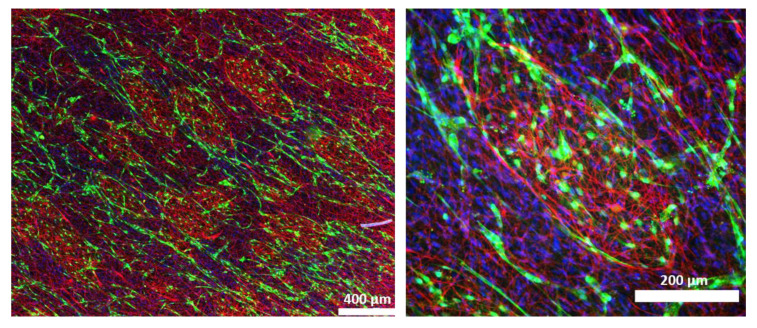
Schwann cells (red) assemble into islands, which are surrounded by lymphatic vessels (green) in co-cultures of LECs, ASCs, and SCs. Green—YFP-LECs, red—SCs stained against S100, blue—cell nuclei.

Interestingly, when SCs were cultivated with LECs and ASCs, SCs sometimes assembled into distinct islands, which were surrounded by lymphatic vascular structures (Figure 6).

To evaluate whether SCs induce apoptosis in a paracrine manner, we cultivated fibrin hydrogels containing either LECs only or a co-culture of LECs and ASCs in SC-conditioned medium (unconditioned medium served as a control). When cultivated in SC-conditioned medium, LECs did not form elongated tube-like structures and grew in distinct clusters, thereby indicating a paracrine effect of SCs on LECs. This effect was less pronounced when LECs were cultivated with ASCs, suggesting that ASCs are able to ameliorate this SC-induced effect (Supplementary Figure S3).

**Figure 7:**
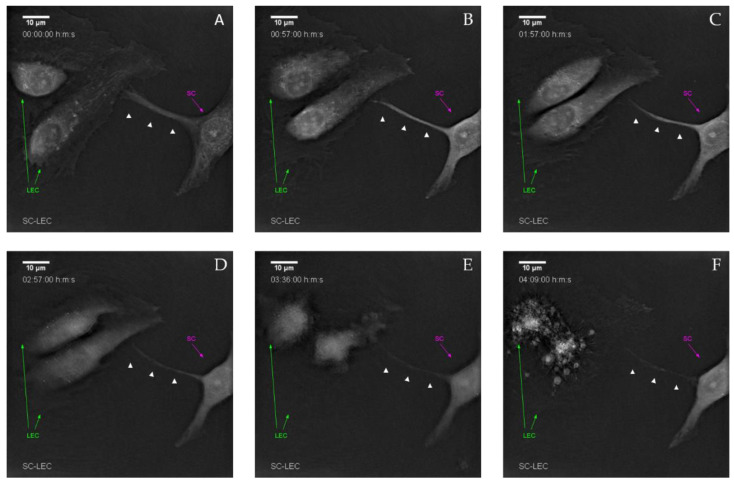
Live cell imaging demonstrates that cell–cell contact induced lymphatic endothelial cell death by Schwann cells. (**A**) Initial cell–cell contact between LECs (green arrow) and SCs (magenta arrow) via filopodia-like protrusions (white arrow heads). (**B**) After initial contact, morphological changes in both LECs and the SC-filopodia-like protrusions become visible. (**C**,**D**) Retraction of both the filopodia-like protrusion and LEC endoplasm. (**E**) Stark retraction and compartmentalization of LECs. (**F**) Formation of LEC-derived apoptotic bodies. Time-lapse shown in each image is the observation time starting 2 h post-seeding.

In order to more closely elucidate how LECs were dying in co-culture with SCs, we performed 2D co-cultures, which were then used for time-lapse imaging, using two different systems: the JuLI (Supplementary Figure S4), as well as the Nanolive system. We observed the induction of apoptotic cell death of LECs over a time period of several hours, resulting in almost complete clearance of LECs in the co-culture within 48 h. The high resolution Nanolive imaging indicates a cell–cell contact induced apoptosis within 5 h of co-culture (Figure 7). In particular, SCs extended filopodia-like protrusions towards LECs, eventually resulting in apoptosis of LECs, as indicated by the formation of LEC-derived apoptotic bodies (Figure 7f). Upon LEC death, SCs again retracted their filopodia-like protrusions. Time-lapse videos are provided in Supplementary Videos S1–S3.

### 3.5. Stimulation of 3D Fibrin Hydrogel Cultures with TNFα Impairs Lymphatic Network Formation

**Figure 8:**
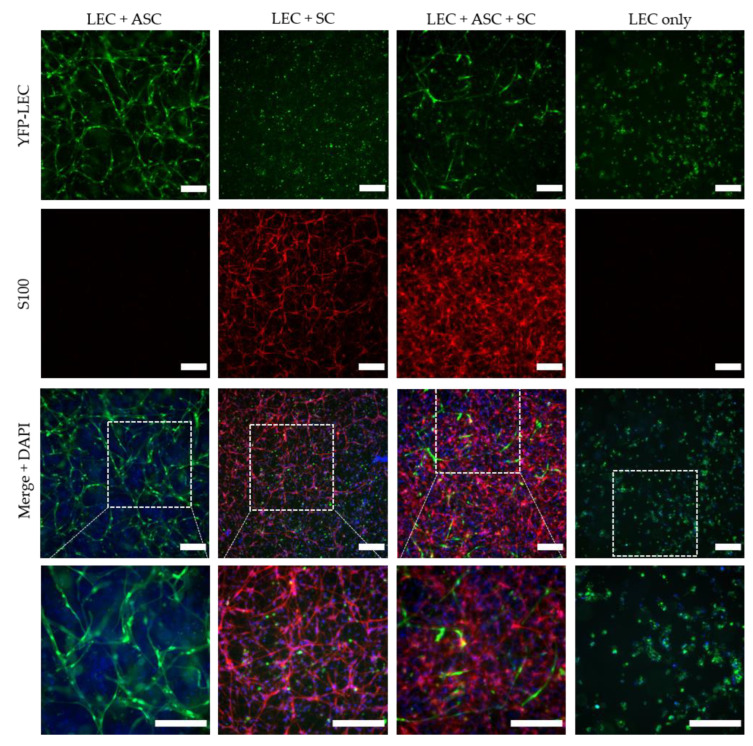
Schwann cells hinder lymphatic vascular network formation in the presence of the pro-inflammatory cytokine TNF-α. LEC + ASC formed elongated and interconnected networks, even when stimulated with TNF-α; whereas the formation of lymphatic vessel-like structures in the LEC + ASC + SC cultures in the presence of TNF-α was hampered. The co-culture of only LECs and SCs did not show the formation of lymphatic tubes. Green—YFP-LECs, red—SCs stained against S100, blue—cell nuclei. Scale bars = 200 µm.

Considering that peripheral nerve regeneration and neuropathies in general are associated with an inflammatory environment, we next aimed to evaluate the influence of the pro-inflammatory cytokine TNF-α on Schwann cell behavior, as well as lymphatic network development. LECs were embedded into 3D fibrin hydrogels, either as monocultures, or together with ASCs, with SCs, or with ASCs and SCs, and stimulated with 10 ng/mL TNF-α for 48 h. Regarding lymphatic network formation, LECs formed more interconnected and dense networks when cultivated with ASCs alone compared to the tri-culture with both ASCs and SCs, whereas in co-culture with SCs and in LEC monocultures, lymphatic vessel development was again completely inhibited (Figure 8). However, compared to 3D cultures that had not been stimulated with TNF-α (Figure 4), lymphatic network formation was generally less pronounced under inflammatory conditions. This might indicate the importance of a compromised lymphatic system during chronic inflammatory neuropathies. The quantification of network parameters showed a significantly smaller area covered with lymphatic networks when LECs were cultivated alone, together with ASCs, or together with ASCs and SCs when stimulated with TNF-α (Figure 5A). Furthermore, stimulation with TNF-α also resulted in fewer junctions, as well as a shorter tubule length in LEC single cultures and co-cultures of LECs, ASCs, and SCs (Figure 5B,C). Comparison of all TNF-α cultures revealed a significantly larger area covered with lymphatic networks, higher numbers of junctions, as well as longer tubule length in the co-culture of LECs with ASCs compared to the tri-culture of LECs with ASCs and SCs, or the co-culture with SCs (Figure 5).

**Figure 9:**
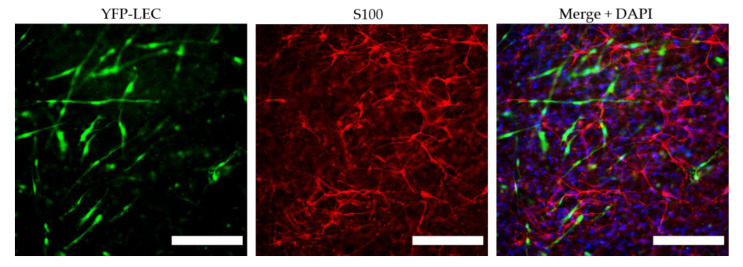
TNF-α induces phenotypical changes in Schwann cells. Stimulation with TNF-α resulted in a morphological change of SCs from a bipolar to a multipolar phenotype, with highly branched protrusions. Green—YFP-LECs, red—SCs stained against S100, blue—cell nuclei. Scale bars = 200 µm.

Interestingly, stimulation with TNF-α also induced changes in SC morphology, especially when cultured together with LECs and ASCs. In the presence of TNF-α, SCs manifested a multipolar morphology, with highly branched protrusions (Figure 9). This phenotype was not observed in unstimulated cultures.

## 4. Discussion

Although a functional lymphatic system for peripheral nerve regeneration is likely important for peripheral nerve repair, the influence of lymphangiogenesis during nerve regeneration has largely not been investigated. In this study, we identified the presence of lymphatic vessels in autologous nerve transplants after segmental median nerve damage, whereas healthy median nerves were void of lymphatic vasculature. Aiming to more closely analyze the interaction between SCs and LECs in vitro, we furthermore found that LEC and SC secretomes did not influence cell migration and proliferation. Moreover, lymphatic microvascular structures were successfully created in SC-embedded 3D fibrin hydrogels in the presence of ASCs as a supporting cell type, whereas SCs inhibited lymphangiogenesis when cultured with LECs only.

Peripheral nerve injuries pose a major clinical concern world-wide, and functional recovery after peripheral nerve injury using nerve grafts is still far from satisfactory. Although it is well established that angiogenesis plays a pivotal role during nerve regeneration [21], the importance of the lymphatic system during peripheral nerve injury and repair has only been hypothesized. The fact that a damaged lymphatic system results in persistent swelling and inflammation due to compromised tissue drainage, both of which are frequently observed after the use of nerve grafts, highlights the urgent need for a more thorough investigation of the lymphatic system in peripheral nerves, in both physiological and pathophysiological settings. Moreover, it has been shown that during embryogenesis, the blood and lymphatic vascular and peripheral nervous system share molecular cues that guide both axonal growth cones, as well as vascular tip cells, during vessel formation, especially class 3 semaphorins [37,38]. Here, we observed lymphatic vessels in autologous nerve grafts after segmental damage of median nerves, while lymphatic vascular structures were absent in healthy median nerves. This might indicate that a functional lymphatic system is required for peripheral nerve repair, but not for the homeostasis of the peripheral nervous system. Observations supporting this theory were published by Meng et al. in 2020, who identified Prox-1 as a key protein, not only in regard to the formation of new lymphatic vessels, but also for nerve regeneration following sciatic crush injury in a rat model. The authors emphasized the importance of this newly formed lymphatic vasculature for the reduction of tissue edema necessary to restore the nerve’s functionality [24]. Based on these observations in vivo, we aimed to elucidate the interaction between SCs and LECs in vitro. First, we performed 2D scratch assay experiments to assess whether the cells’ secretome affects migration of LECs and SCs after creating a cell-free area by scratching confluent LEC and SC monolayers; however, there only seemed to be a slight trend of cell migration being slower when cultivated in its respective conditioned medium compared to unconditioned medium, as well as the other cells’ secretome. Since SC-conditioned medium did not impact LEC migration, we continued to analyze the effect of SCs on lymphatic vessel formation through direct contact; first in 2D experiments and subsequently in 3D fibrin cultures. ASCs were used as a supporting cell type for lymphatic vessel formation, since we had previously shown that ASCs promote lymphatic tube formation in 2D as well as 3D cultures [33], and it has also been reported that lymphangiogenesis is supported by ASCs via secretion of factors such as angiopoietin-1, VEGF-A and -D, fibroblast growth factor 2 and hepatocyte growth factor [39]. Within a period of only 7 days, LECs co-cultured with ASCs and SCs formed elongated and interconnected networks that were, however, less dense than those of the positive control (LEC + ASC). To further investigate the reduced lymphatic network density in tri-cultures of LECs, ASCs, and SCs, the addition of another cell type into the positive control hydrogels (LEC + ASC) would be important, to determine whether this effect is related to an increased cell density within the hydrogel. Additionally, Landau et al. recently reported that cultivation of LECs with fibroblasts failed to induce lymphatic vessel formation, and they hypothesized that the inhibition of vessel formation was due to the production of dense collagen layers and excessive tension present in these cultures [40]. Consequently, to screen for the presence of different extracellular matrix (ECM) proteins, and the potential differences thereof between the different cultures, would allow identifying a correlation between ECM secretion and lymphangiogenesis.

Interestingly, when LECs were cultivated with SCs only, not even primitive tube-like structures could be observed in 3D fibrin hydrogels. Furthermore, LECs showed an untypical round phenotype, indicating that SCs might induce apoptosis of LECs. In fact, SCs appear to induce apoptosis in LECs via the extension of filopodia-like protrusions in a direct 2D co-culture. Furthermore, we observed an altered LEC morphology when cultivated in SC-conditioned medium, resulting in a less elongated morphology, cell clustering, and the absence of even primitive elongated tube-like structures; indicating, at least partly, a paracrine effect of SCs on LECs. The fact that this effect was nevertheless not as prominent as in a direct co-culture of LECs with SCs, on the one hand, indicates that it was rather a combined paracrine and juxtacrine effect of SCs on LECs, and on the other hand, that ASCs as a supporting cell type were able to ameliorate this effect. Consequently, to identify the molecular mechanisms involved in this process, the analysis of differential expression of pro- versus anti-lymphangiogenic genes and proteins in triple-cultures of LECs, ASCs, and SCs compared to co-cultures of LECs and ASCs would be of great relevance. For example, Huang et al. showed that SCs produce anti-angiogenic molecules and identified high levels of tissue inhibitor of metalloproteinase-2 (TIMP-2), as well as low levels of pigment epithelium-derived factor (PEDF)—both known inhibitors of angiogenesis—in SC supernatants [41,42]. However, in contrast to our observations, they also observed an inhibitory effect of SC-conditioned medium, specifically on blood endothelial cell proliferation and migration [41,42]. In another study, the same group identified secreted protein acidic and rich in cysteine (SPARC) as another essential anti-angiogenic molecule expressed and secreted by SCs, which not only inhibited blood endothelial cell migration but also induced apoptosis in vitro, and furthermore suppressed angiogenesis in vivo, the effects of which could be fully reversed by the addition of neutralizing anti-SPARC antibody [43]. Moreover, SPARC seemed to inhibit lymphangiogenesis in ovarian cancer through reduced VEGF-C and -D expression [44]. These findings suggest that several factors expressed by SCs act synergistically to inhibit the formation of vasculature. In accordance with this, we observed an increased amount of SPARC present in the supernatant of LEC + SC fibrin hydrogels on day 7 compared to the other hydrogel groups (data not shown), indicating that the anti-lymphangiogenic effect might be, at least in part, caused by the secretion of SPARC.

Another interesting observation was the assembly of SCs into distinct islands, which were enclosed by lymphatic vessels. Hence, to analyze cell–cell interactions between SCs and LECs might provide important insights into the role of lymphatic vessels for SC guidance, as well as the influence of SC organization on lymphatic vessel assembly.

Since peripheral nerve injuries and neuropathies are accompanied by inflammation, we aimed to assess the influence of the pro-inflammatory cytokine TNF-α on lymphatic network formation, as well as on SC behavior. Stimulation with TNF-α, not only resulted in reduced numbers of lymphatic vessels present in all cultures, but also induced an altered SC morphology, with multiple highly branched and very long protrusions, especially when cultured with ASCs and LECs.

Although we here, for the first time, described the interaction between SCs and lymphatics in vitro, our study has two main limitations. First, we used a physiologically non-representative combination of cells in our in vitro experiments; i.e., the use of LECs and ASCs derived from human material together with SCs isolated from rat nerves, due to the limited availability of human nerve tissue. Hence, it would be an interesting option to perform the experiments presented in our study with SCs derived from human-induced pluripotent stem cells, to confirm that the results are reproducible in a fully human system. Second, this is a primarily descriptive study, lacking further analyses to elucidate underlying mechanisms. Apart from the analysis of pro- and anti-lymphangiogenic genes already mentioned above, in particular the gene expression profiles of cells in the different fibrin hydrogel cultures, to assess (i) the influence of LECs on SC phenotype, and (ii) the influence of stimulation with TNF-α on SC phenotype, remain to be elucidated.

Overall, our study highlights the feasibility of engineering lymphatic vessels in the presence of SCs, which paves the way for the generation of more complex tissue engineered constructs, comprising Schwann cells, neuronal cells, as well as blood and lymphatic endothelial cells. Such peripheral nerve-like tissue with separate blood and lymphatic vascular networks are desperately needed as biomimetic in vitro models, to adequately study neuropathies, the neurotoxic effects of drugs, as well as peripheral nerve regeneration. Furthermore, we for the first time described that SCs induce the apoptotic cell death of LECs via extended filopodia-like protrusions, within only 5 h of co-culture in vitro, eventually resulting in complete clearance of LECs within 48 h. Of note, this interesting observation might provide a possible explanation for the lack of lymphatic vessels in the endoneurium of healthy peripheral nerves. However, at the same time the underlying mechanisms of SC-induced apoptosis of LECs need to be further elucidated, in order to manipulate lymphangiogenesis, thereby possibly enhancing peripheral nerve repair. This might pave the way for the establishment of novel therapeutic approaches for peripheral nerve injuries.

## Data Availability

All data underlying this study are included in the manuscript and the supplementary material. Original data will be provided upon request.

## Supplementary Materials

The following supporting information can be downloaded at: https://www.mdpi.com/article/10.3390/biom12060820/s1, Figure S1: Characterization of primary rat Schwann cell cultures; Figure S2: Representative images of the Schwann cell and lymphatic endothelial cell scratch assay experiments; Figure S3: Lymphatic network formation is hindered when cultivated in Schwann cell-conditioned medium, Figure S4: Live cell imaging of YFP-LEC and SC co-culture with the JuLI Smart fluorescent analyzer offers visual confirmation of the diminishing number of YFP-LECs present in the selected field of view, over 48 hours; Table S1: Detailed statistics report of Angiotool network quantification; Video S1: Time-lapse imaging of YFP-LEC and SC co-culture with the high resolution Nanolive imaging system; Video S2: Time-lapse imaging of YFP-LEC and SC co-culture with the JuLi imaging system (GFP channel); Video S3: Time-lapse imaging of YFP-LEC and SC co-culture with the JuLi imaging system (overlay of brightfield + GFP channel); File S1: Detailed performance report of the automated quantification of lymphatic vessels with the IKOSA platform.
