# Supplementary material for: Occurrence of Lymphangiogenesis in Peripheral Nerve Autografts Contrasts Schwann Cell-Induced Apoptosis of Lymphatic Endothelial Cells In Vitro
Source: Biomolecules. 2022 Jun 12;12(6):820. doi: 10.3390/biom12060820 (PMC9221261; doi:10.3390/biom12060820)
Supplement: Supplementary file 1 [file biomolecules-12-00820-s001.zip › Supplementary Figures + Table.pdf]

Supplementary Figures

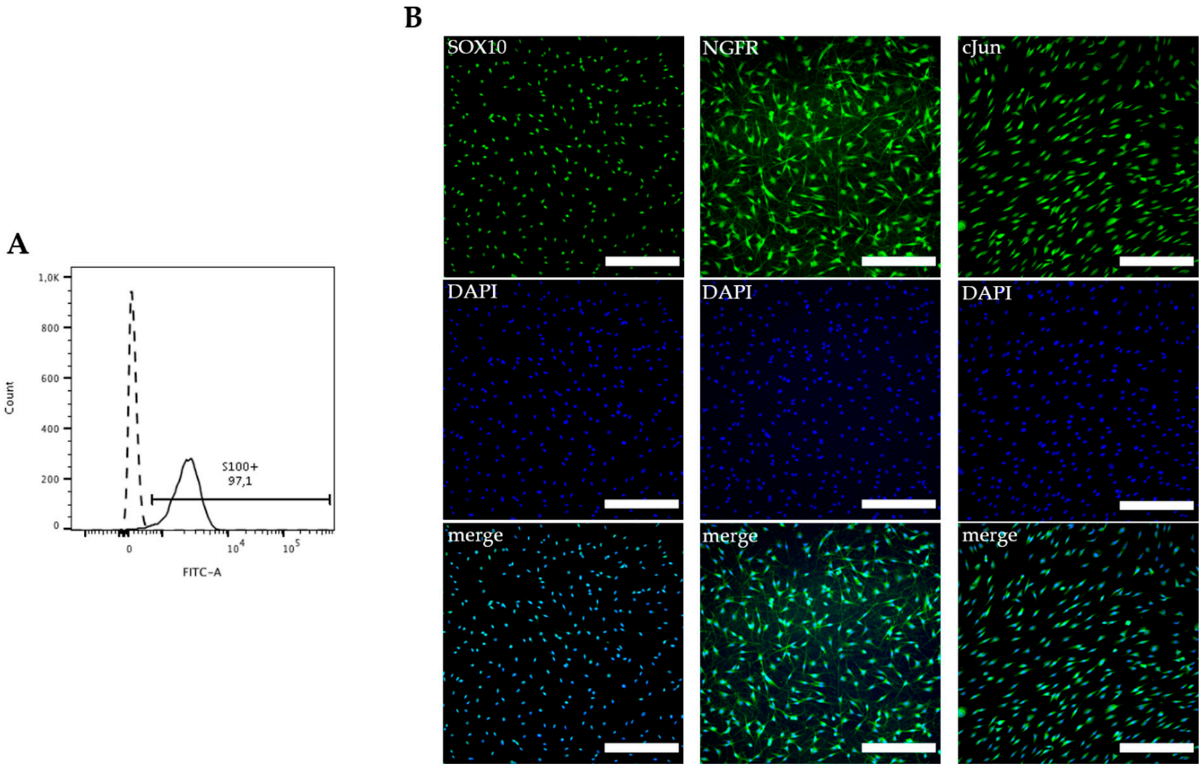

Supplementary Figure S1: Characterization of primary rat Schwann cell cultures. A) Representative flow cytometry histogram showing a high purity of >97% S100+ Schwann cells. Dashed line represents secondary antibody control, solid line shows S100 stained cells. B) Representative immunofluorescence images of Schwann cells stained against SOX10, NGFR and cJun. A DAPI counterstain was performed to visualize nuclei. Scale bars = 200 µm.

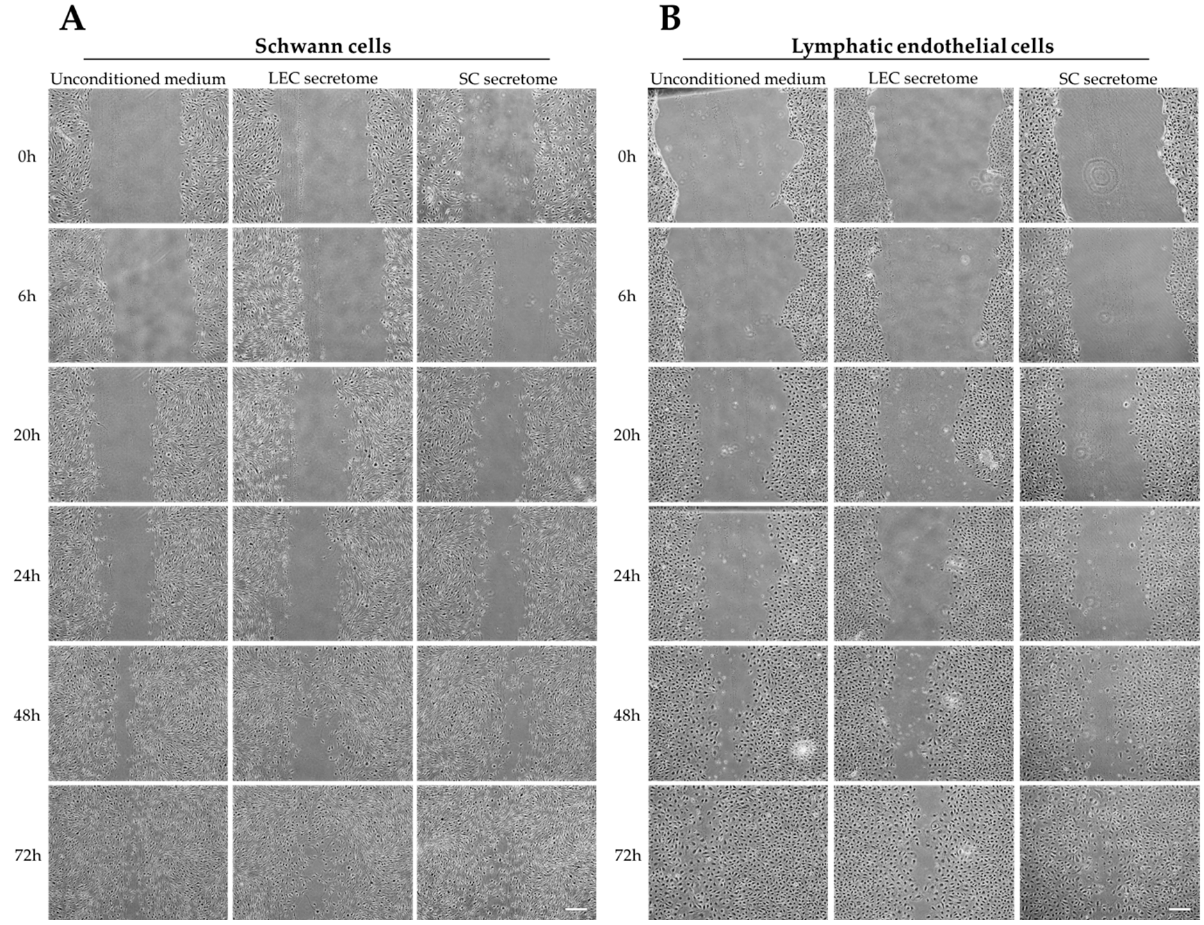

Supplementary Figure S2: Representative images of the Schwann cell and lymphatic endothelial cell scratch assay experiments. SCs (A) and LECs (B) were grown until confluency and their conditioned media were collected 24 hours before the experiment. Scratches were created on cell monolayers and cell migration in either unconditioned medium or conditioned medium from LEC or SC was monitored over a total of 72 hours. Scale bars = 300 μm.

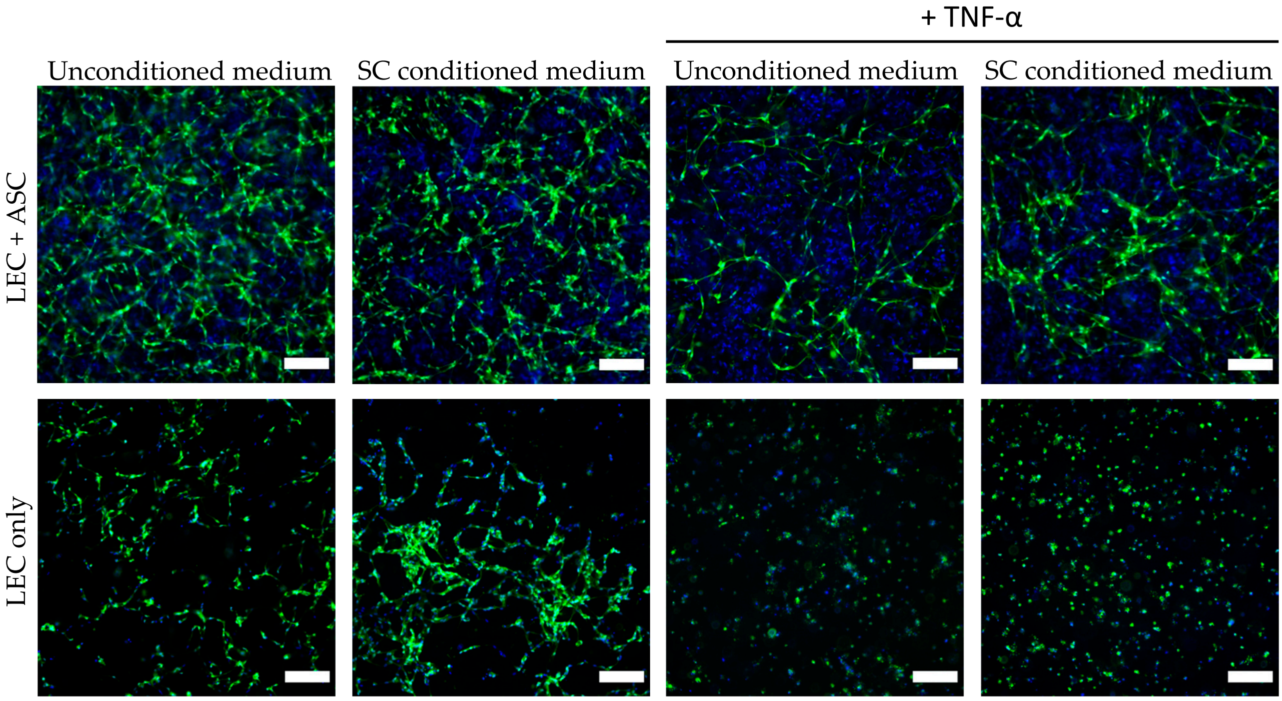

Supplementary Figure S3: Lymphatic network formation is hindered when cultivated in Schwann cell-conditioned medium. LECs and ASCs or LECs only were cultivated in fibrin hydrogels for 7 days containing either unconditioned medium or Schwann-cell conditioned medium. LECs failed to form tube-like structures when cultivated in SC-conditioned medium and furthermore clustered together. In contrast, this effect was less pronounced when LECs were cultivated with ASCs. Green – LECs. Scale bars = 200 µm.

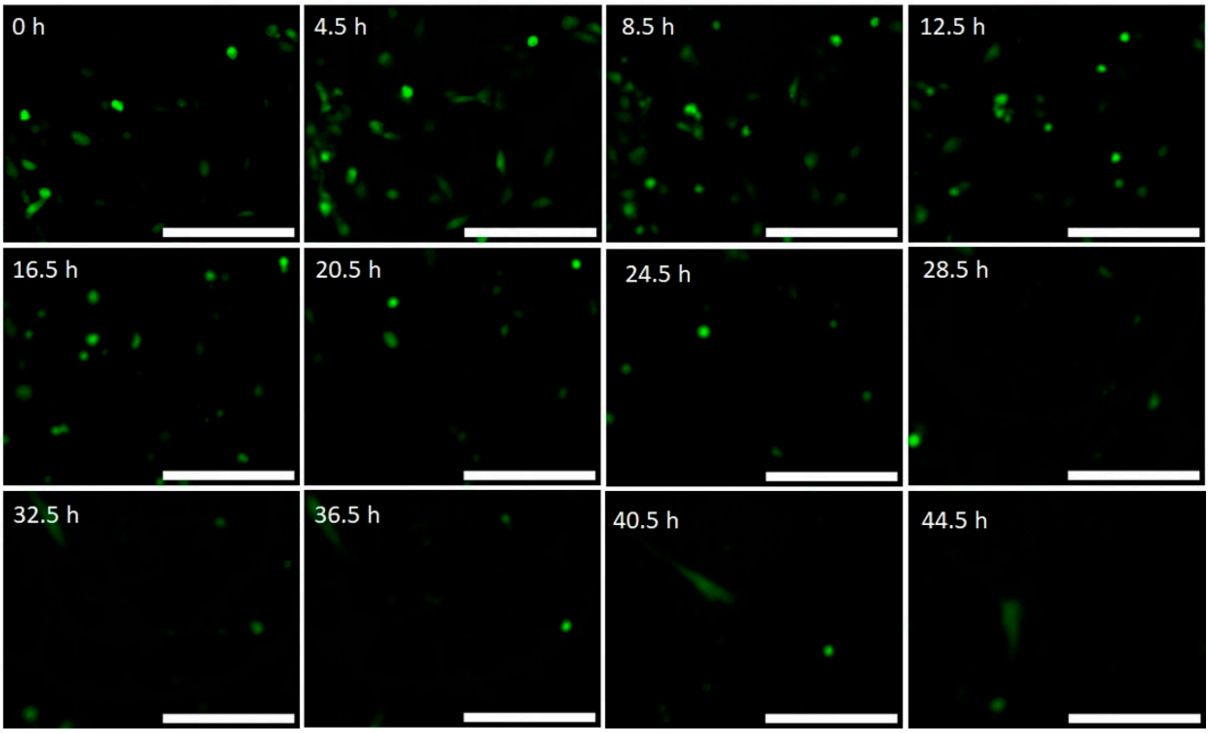

Supplementary Figure S4: Live cell imaging of YFP-LEC and SC co-culture with the JuLI Smart fluorescent analyzer offers visual confirmation of the diminishing number of YFP-LECs present in the selected field of view, over 48 hours. Exhibited in this figure are selected images collected during the 48-hour time period showcasing YFP-LECs exhibiting green fluorescence. SCs are not fluorescently labeled and are therefore not visualized here. Scale bars = 100 µm.

Supplementary Tables:

Supplementary Table S1: Detailed statistics report of Angiotool network quantification.

	Tubule length rel. pos. ctrl					No of Junctions rel. pos. ctrl					Vessels percentage area						
Tukey's multiple comparisons test	Mean Diff,	95,00% CI of diff,	Below threshold?	Summary	Adjusted P Value	Mean Diff,	95,00% CI of diff,	Below threshold?	Summary	Adjusted P Value	Mean Diff,	95,00% CI of diff,	Below threshold?	Summary	Adjusted P Value	n1	n2
LEC + SC + ASC vs. LEC + SC + ASC + TNF	49.81	32,70 to 66,92	Yes	****	<0,0001	42.54	18,84 to 66,24	Yes	****	<0,0001	21.19	15,72 to 26,66	Yes	****	<0,0001	24	23
LEC + SC + ASC vs. LEC + SC	73.57	49,64 to 97,51	Yes	****	<0,0001	64.2	31,05 to 97,36	Yes	****	<0,0001	28.16	20,51 to 35,81	Yes	****	<0,0001	24	8
LEC + SC + ASC vs. LEC + SC + TNF	67.22	44,30 to 90,14	Yes	****	<0,0001	60.55	28,80 to 92,29	Yes	****	<0,0001	26.54	19,22 to 33,87	Yes	****	<0,0001	24	9
LEC + SC + ASC vs. LEC + ASC	-20.68	-37,13 to -4,230	Yes	**	0.0041	-34.47	-57,25 to -11,69	Yes	***	0.0002	-9.524	-14,78 to -4,268	Yes	****	<0,0001	24	27
LEC + SC + ASC vs. LEC + ASC + TNF	-4.885	-21,33 to 11,56	No	ns	0.984	-16.39	-39,17 to 6,392	No	ns	0.3482	-1.525	-6,934 to 3,883	No	ns	0.9882	24	27
LEC + SC + ASC vs. LEC only	18.61	-4,310 to 41,52	No	ns	0.2038	14.84	-16,91 to 46,58	No	ns	0.8362	6.573	-0,7503 to 13,90	No	ns	0.1129	24	9
LEC + SC + ASC vs. LEC only + TNF	72.61	43,79 to 101,4	Yes	****	<0,0001	61.22	21,29 to 101,1	Yes	***	0.0002	27.56	18,35 to 36,77	Yes	****	<0,0001	24	5
LEC + SC + ASC + TNF vs. LEC + SC	23.76	-0,3009 to 47,83	No	ns	0.0556	21.66	-11,67 to 55,00	No	ns	0.4835	6.966	-0,7246 to 14,66	No	ns	0.1062	23	8
LEC + SC + ASC + TNF vs. LEC + SC + TNF	17.41	-5,642 to 40,46	No	ns	0.2867	18.01	-13,92 to 49,94	No	ns	0.662	5.353	-2,013 to 12,72	No	ns	0.3349	23	9
LEC + SC + ASC + TNF vs. LEC + ASC	-70.49	-87,12 to -53,85	Yes	****	<0,0001	-77.01	-100,1 to -53,97	Yes	****	<0,0001	-30.72	-36,03 to -25,40	Yes	****	<0,0001	23	27
LEC + SC + ASC + TNF vs. LEC + ASC + TNF	-54.69	-71,33 to -38,06	Yes	****	<0,0001	-58.93	-81,98 to -35,89	Yes	****	<0,0001	-22.72	-28,18 to -17,25	Yes	****	<0,0001	23	27
LEC + SC + ASC + TNF vs. LEC only	-31.2	-54,26 to -8,151	Yes	**	0.0014	-27.7	-59,63 to 4,227	No	ns	0.1402	-14.62	-21,98 to -7,251	Yes	****	<0,0001	23	9
LEC + SC + ASC + TNF vs. LEC only + TNF	22.8	-6,129 to 51,73	No	ns	0.2363	18.68	-21,40 to 58,75	No	ns	0.8382	6.369	-2,876 to 15,61	No	ns	0.405	23	5
LEC + SC vs. LEC + SC + TNF	-6.354	-34,84 to 22,14	No	ns	0.9972	-3.659	-43,12 to 35,80	No	ns	>0,9999	-1.613	-10,72 to 7,491	No	ns	0.9994	8	9
LEC + SC vs. LEC + ASC	-94.25	-117,9 to -70,65	Yes	****	<0,0001	-98.68	-131,4 to -65,99	Yes	****	<0,0001	-37.68	-45,22 to -30,14	Yes	****	<0,0001	8	27
LEC + SC vs. LEC + ASC + TNF	-78.46	-102,1 to -54,86	Yes	****	<0,0001	-80.6	-113,3 to -47,91	Yes	****	<0,0001	-29.68	-37,33 to -22,03	Yes	****	<0,0001	8	27
LEC + SC vs. LEC only	-54.97	-83,46 to -26,48	Yes	****	<0,0001	-49.37	-88,83 to -9,906	Yes	**	0.0044	-21.58	-30,69 to -12,48	Yes	****	<0,0001	8	9
LEC + SC vs. LEC only + TNF	-0.9631	-34,39 to 32,46	No	ns	>0,9999	-2.988	-49,29 to 43,31	No	ns	>0,9999	-0.5965	-11,28 to 10,08	No	ns	>0,9999	8	5
LEC + SC + TNF vs. LEC + ASC	-87.9	-110,5 to -65,33	Yes	****	<0,0001	-95.02	-126,3 to -63,76	Yes	****	<0,0001	-36.07	-43,28 to -28,86	Yes	****	<0,0001	9	27
LEC + SC + TNF vs. LEC + ASC + TNF	-72.11	-94,67 to -49,54	Yes	****	<0,0001	-76.94	-108,2 to -45,68	Yes	****	<0,0001	-28.07	-35,39 to -20,75	Yes	****	<0,0001	9	27
LEC + SC + TNF vs. LEC only	-48.61	-76,25 to -20,97	Yes	****	<0,0001	-45.71	-83,99 to -7,426	Yes	**	0.008	-19.97	-28,80 to -11,14	Yes	****	<0,0001	9	9
LEC + SC + TNF vs. LEC only + TNF	5.391	-27,31 to 38,09	No	ns	0.9996	0.6706	-44,63 to 45,97	No	ns	>0,9999	1.016	-9,434 to 11,47	No	ns	>0,9999	9	5
LEC + ASC vs. LEC + ASC + TNF	15.79	-0,1648 to 31,75	No	ns	0.0546	18.08	-4,023 to 40,18	No	ns	0.196	7.999	2,742 to 13,26	Yes	***	0.0002	27	27
LEC + ASC vs. LEC only	39.28	16,72 to 61,85	Yes	****	<0,0001	49.31	18,05 to 80,57	Yes	****	<0,0001	16.1	8,886 to 23,31	Yes	****	<0,0001	27	9
LEC + ASC vs. LEC only + TNF	93.29	64,74 to 121,8	Yes	****	<0,0001	95.69	56,15 to 135,2	Yes	****	<0,0001	37.08	27,96 to 46,21	Yes	****	<0,0001	27	5
LEC + ASC + TNF vs. LEC only	23.49	0,9250 to 46,06	Yes	*	0.035	31.23	-0,02978 to 62,49	No	ns	0.0504	8.099	0,7752 to 15,42	Yes	*	0.0193	27	9
LEC + ASC + TNF vs. LEC only + TNF	77.5	48,95 to 106,0	Yes	****	<0,0001	77.61	38,07 to 117,1	Yes	****	<0,0001	29.09	19,88 to 38,30	Yes	****	<0,0001	27	5
LEC only vs. LEC only + TNF	54	21,30 to 86,71	Yes	****	<0,0001	46.38	1,082 to 91,68	Yes	*	0.0407	20.99	10,54 to 31,44	Yes	****	<0,0001	9	5
