# Supplementary material for: Occurrence of Lymphangiogenesis in Peripheral Nerve Autografts Contrasts Schwann Cell-Induced Apoptosis of Lymphatic Endothelial Cells In Vitro
Source: Biomolecules. 2022 Jun 12;12(6):820. doi: 10.3390/biom12060820 (PMC9221261; doi:10.3390/biom12060820)
Supplement: Supplementary file 1 [file biomolecules-12-00820-s001.zip › Supplementary File S1.pdf]

I K O S A

Training Report

Application name
OEJ Lymphatics V2

October 20, 2021

This report provides a summary of your IKOSA AI training and supports you with the assessment of the performance of your trained application. An overview is given of the images used for training and validation. To evaluate the accuracy, your trained application performed an automated analysis of the validation images and generated results. Those results were compared to the ground truth annotations (your manual annotations).

We provide quantitative results in chapter 4.1. Qualitative results in form of visualizations are provided as separate image files in addition to this PDF report. A description for them can be found in chapter 4.2. You can use it to assess whether the result meets your requirements or if you need to annotate more training images to achieve the desired accuracy.

If you have any questions regarding your training, please send us an email at **support@ikosa.ai**.

Please copy and add your training ID `af4b5597-cc74-49f6-b0c4-c04452b87e06` in your email.

We wish you successful applications! Your IKOSA AI team.

Table of Contents

1. Training Type and Time
2. Projects and Labels
3. Training Images
4. Validation Images
    4.1. Quantitative Results
    4.2. Qualitative Results
5. Appendix
6. Contact and Support

1. Training Type and Time

Your trained application performs a **segmentation** of image regions/objects that look similar to the ones that have been annotated and labelled manually in the training images.

For segmentation, the AI of your trained application predicts for each pixel of an image if it belongs to a certain label with a specific confidence. Then thresholding is applied, i.e. if the confidence is higher than 50% for a specific label, this label is assigned to the pixel. If the confidence is less than or equal to 50%, the pixel is treated as background. Further post-processing removes very small objects of less than 10 pixels area.

Training time: 12 minutes, 32 seconds

2. Project and Labels

Your application was trained with images in project "**Rat Podoplanin**".

Project ID: `3dccedd7-bcbc-4901-9b7b-7d316e1ec92f`

The following labels were included in the training:

- lymphatic vessel: Lymph Vessel

3. Training Images

For the training of your application, **10 training images (including 233 ROI)** were used. The following table lists all training images and ROI included in the training together with the included annotations.

Images with Annotations

Image Name	ROI	Annotations
AC5855_rPodo_Scan1.tif	10	21
AC5987_Podo_Scan1.qptiff	33	82
AC6238_rPodo_Scan1.tif	2	12
AC6263_rPodo_Scan1.tif	4	54
AC6323_rPodo_Scan1.tif	22	117
**Total: 5 images with annotations**	**71**	**286**

Images without Annotations

Image Name	ROI	Annotations
AC5876_Podo_Scan1.qptiff	46	-
AC5990_Podo_Scan1.qptiff	53	-
AC6243_rPodo_Scan1.tif	22	-
AC6278_rPodo_Scan1.tif	2	-
AC6303_rPodo__Scan1.tif	39	-
**Total: 5 images without annotations**	**162**	**-**

4. Validation Images

For the performance evaluation of your application, **3 validation images (including 42 ROI)** were used. These images were **selected randomly** from all included images (effectively using 77% for training and 23% for validation). When starting trainings with exactly the same images and ROI, the same images are used for training and validation, respectively. The following tables list all validation images and ROI included in the evaluation together with the included annotations per label.

The subsequent sections provide quantitative and qualitative results of the automated analysis with your trained application on the validation images.

Images with Annotations

Image Name	ROI	Annotations
AC5873_Podo_Scan1.qptiff	32	87

Image Name	ROI	Annotations
AC5888_Podo_Scan1.qptiff	5	11
AC5903_Podo_Scan1.qptiff	5	7
**Total: 3 images with annotations**	**42**	**105**

Images without Annotations

Image Name	ROI	Annotations
**Total: 0 images without annotations**	**0**	**-**

4.1. Quantitative Results

In the following section, tables with quantitative results for all validation images and all available labels are presented. We provide the measures **Dice Coefficient, Precision** and **Recall** overall as well as for each image and ROI. An explanation of the different metrics can be found at the end of the report in chapter 5.1 along the interpretation of the table row values in chapter 5.2.

Lymph Vessel

Overall Label Peformance	Labeled Annotations	Dice Coefficient	Precision [%]	Recall [%]
**All images and ROI**	**105**	**0.86**	**91.54**	**80.74**

Images

Image Name	ROI	Labeled Annotations	Dice Coefficient	Precision [%]	Recall [%]
**Total: 0 images**		**-**	**nan**	**nan**	**nan**

ROI

Image Name	ROI	Labeled Annotations	Dice Coefficient	Precision [%]	Recall [%]
AC5873_Podo_Scan1.qptiff	all ROI (32)	87	0.87	92.17	82.61
	ROI-01	2	0.88	97.65	80.31
	ROI-02	2	0.83	93.09	74.64
	ROI-03	6	0.83	98.35	71.61
	ROI-04	4	0.86	96.73	76.85
	ROI-05	2	0.91	99.86	84.14
	ROI-06	3	0.91	93.69	87.80
	ROI-07	1	0.91	98.35	84.40
	ROI-08	4	0.79	96.19	67.16
	ROI-09	3	0.82	89.74	74.86
	ROI-10	1	0.82	96.54	70.82
	ROI-11	2	0.88	94.55	81.84
	ROI-12	1	0.93	89.64	95.67

Image Name	ROI	Labeled Annotations	Dice Coefficient	Precision [%]	Recall [%]
	ROI-13	4	0.87	96.27	78.82
	ROI-14	4	0.86	88.99	83.14
	ROI-15	4	0.75	82.80	69.15
	ROI-16	1	0.92	96.04	88.48
	ROI-17	1	0.93	94.89	90.76
	ROI-18	1	0.90	93.44	87.69
	ROI-19	4	0.89	96.58	83.13
	ROI-20	1	0.97	100.00	93.94
	ROI-21	8	0.87	90.35	83.66
	ROI-22	2	0.79	72.13	88.00
	ROI-23	4	0.89	91.80	85.98
	ROI-24	1	0.87	95.83	79.31
	ROI-25	1	0.59	44.38	86.59
	ROI-26	4	0.79	77.31	81.42
	ROI-27	2	0.87	96.32	79.29
	ROI-28	5	0.93	89.28	97.24
	ROI-29	1	0.81	72.05	92.80
	ROI-30	2	0.84	73.17	98.36
	ROI-31	5	0.92	91.08	93.08
	ROI-32	1	0.60	43.64	96.00
AC5888_Podo_Scan1.qptiff	all ROI (5)	11	0.85	83.54	87.43
	ROI-01	5	0.80	80.05	80.05
	ROI-02	2	0.91	97.73	84.87
	ROI-03	1	0.75	85.71	66.67
	ROI-04	2	0.88	82.98	94.35
	ROI-05	1	0.78	85.71	72.00
AC5903_Podo_Scan1.qptiff	all ROI (5)	7	0.72	95.28	57.82
	ROI-01	1	0.86	94.58	78.92
	ROI-02	2	0.40	96.13	25.00
	ROI-03	2	0.91	96.39	86.57
	ROI-04	1	0.93	95.00	90.48
	ROI-05	1	0.84	90.91	77.67
**Total: 3 images**	**42**	**105**	**0.86**	**91.54**	**80.74**

4.2. Qualitative Results

Qualitative results for all validation images and ROI are presented as visualizations in separate image files in addition to this PDF report. For each validation image or ROI, the following visualizations are included:

- Overviews

- Image and ROI Performance
- Confidences

Overviews

If a particular validation image `<image-name>` includes one or multiple ROI, an overview visualization of the ROI location(s) within the image is included.

File name: `rois_visualization/<image-name>_rois_visualization.jpg`

Image and ROI Performance

A visualization showing the performance of your trained application for a specific label `<label-name>` on the image or the ROI is located in the `validation_vis` folder.

File names:

- `validation_vis/<label-name>_<image-name>.jpg`
  visualization of image `<image-name>` that contains no ROI
- `validation_vis/<label-name>_<image-name>_<roi-id>.jpg`
  visualization for ROI `<roi-id>` in image `<image-name>`

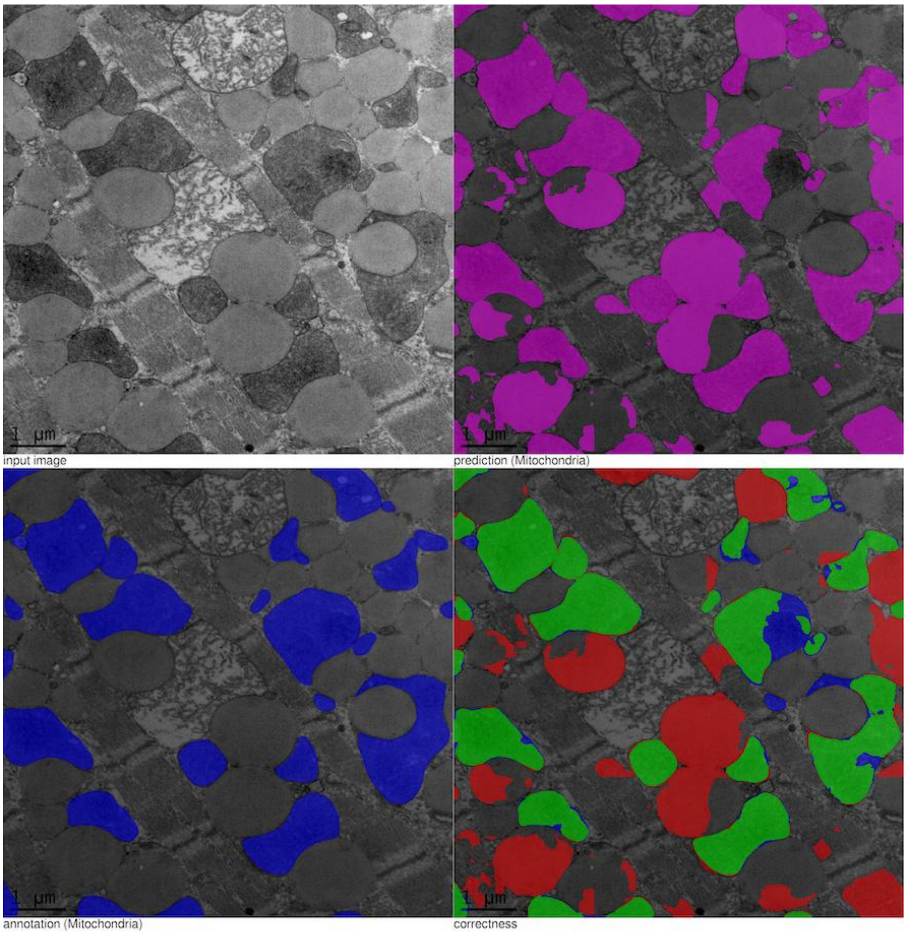

This visualization includes:

- **Left upper image ("input image")** shows the input image.
- **Right upper image ("prediction (<label-name>)")** shows an overlay of the automated prediction of your trained application on the input image.
- **Left lower image ("annotation (<label-name>)")** shows an overlay of the ground truth annotations (your manual annotations) on the input image.

- **Right lower image ("correctness")** visualizes the correctness of the automated prediction (compared to manual annotations):
  - **True-positive** areas are shown in **green**. These are all areas that are segmented by your trained application correctly.
  - **False-positive** areas are shown in **red**. These are all areas that are segmented by your trained application, but were not annotated by you manually.
  - **False-negative** areas are shown in **blue**. These are all areas that were annotated by you manually, but are not segmented by your trained application.

Confidences

We include a visualization of your trained application's prediction confidence for each validation image/ROI and trained label as an estimate of its certainty. You can use the visualizations to determine, where the model still has difficulties determining the correct label.

File names:

- `validation_vis/<label-name>_<image-name>_conf.jpg`
  visualization of image `<image-name>` that contains no ROI
- `validation_vis/<label-name>_<image-name>_<roi-id>_conf.jpg`
  visualization for ROI `<roi-id>` in image `<image-name>`

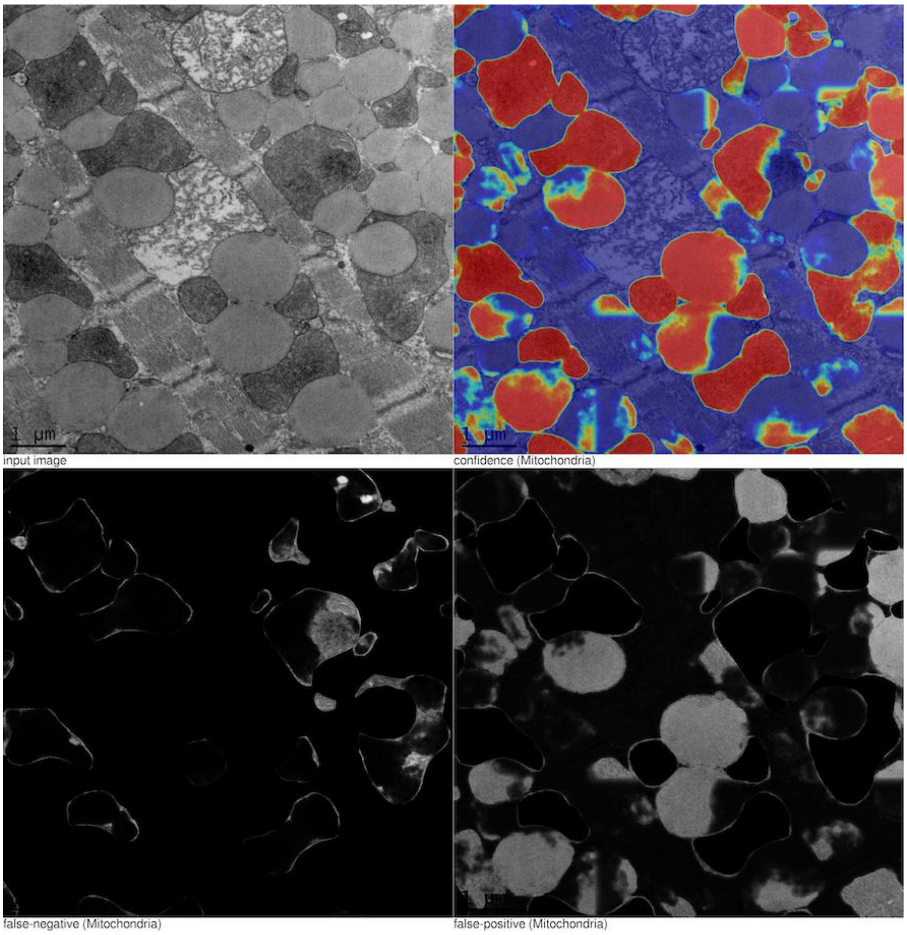

This visualization includes:

- **Left upper image ("input image")** shows the input image.
- **Right upper image ("confidence (<label-name>)")** image shows an overlay of the trained application's confidence for label `<label-name>` as heatmap. It is rather confident that it detects this particular label in regions colored towards the red spectrum (1). Similarly, it is rather confident that image regions towards the blue spectrum (0) *do not* belong to this particular label. The confidence in areas shown in cyan to yellow is lower, indicating a less certain decision.

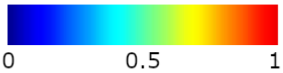

- **Left lower image ("false-negative (<label-name>)")** shows a mask of false-negative regions. The confidence heatmap for label `<label-name>` masks all image regions in black that were segmented correctly. All visible (non-black) areas were annotated by you manually, but were not segmented by your trained application. The more visible an area, the more confident the application is that it *does not* belong the desired label.
- **Right lower image ("false-positive (<label-name>)")** shows a mask of false-positive regions. The confidence heatmap for label `<label-name>` masks all image regions in black that were segmented correctly. All visible (non-black) areas were segmented by your trained application, but were not annotated by you manually. The more visible an area, the more confident the model is that it belongs to the desired label.

Regions you see in the false-positive and false-negative visualizations were not (yet) recognized correctly by your trained application, essentially indicating that it did not yet sufficiently learn the features of the desired label. In an ideal application, both images are black and don't show any regions. In general, you can improve the performance by training on more images/ROI or for a longer time until it becomes more confident in its decisions.

Please Note

- ROI that are completely outside of images are not included in training, however, they are still listed in corresponding tables.
- The `<roi-id>` is generated automatically according to the creation date of the ROI. The location of a ROI with a specific `<roi-id>` within an image can be seen in the file `rois_visualizations/<image-name>_rois_visualization.jpg`. ROI that are completely outside of the image are not shown in this file.
- All visualizations are downscaled to 25 megapixels (MP) if the visualization in the original image size would be larger than 25 MP.

5. Appendix

5.1. Quantitative Result Metrics

Metrics for semantic segmentation are computed per label individually from the confusion matrix. It contains the count of ground truth and predicted pixel labels, i.e. true positives, false positives, true negatives, and false negatives. All metrics are computed unweighted for each ROI, image, and as a total for each label in a bottom-up fashion and do not represent an aggregation such as average or median values.

Dice Coefficient

Dice Coefficient is a measure for the similarity between the results of your trained application and the ground truth annotation (your manual annotation) and is calculated as

$$\text{Dice Coefficient} = \frac{2|X \cap Y|}{|X| + |Y|} = \frac{2 \cdot \text{True Positive Area}}{\text{Predicted Area} + \text{Ground Truth Area}},$$

where X is the ground truth annotation and Y the result of your application. It ranges from 0 to 1, where 0 means that there is no overlap between result and annotation and 1 means that the result is equal to the ground truth annotation.

The closer the value to 1, the better your trained application performs.

Please note: If the image does not contain any ground truth annotation or prediction area at all, the Dice Coefficient becomes *nan* (Not A Number).

Precision

Precision (positive predictive value) shows the percentage of the predicted area that overlaps with the ground truth and is calculated as

$$\text{Precision} = 100 \cdot \frac{\text{True Positive Area}}{\text{Predicted Area}}.$$

The closer the value to 100%, the better your trained application performs.

Please note: If the image does not contain any prediction area at all, the Precision becomes *nan* (Not A Number).

Recall

Recall (Sensitivity, true positive rate) shows the percentage of the ground truth area that is predicted by the application and is calculated as

$$\text{Recall} = 100 \cdot \frac{\text{True Positive Area}}{\text{Ground Truth Area}}.$$

The closer the value to 100%, the better your trained application performs.

Please note: If the image does not contain any ground truth annotation at all, the Recall becomes *nan* (Not A Number).

5.2. Table Interpretation

Value representations:

- **"nan"**
  if values cannot be computed (see chapter 5.1)
- **"-" (dash)**
  if an image does not contain any ROI, or if an image or ROI does not contain any labeled annotations

Edge Cases

Certain edge cases may occur as side effect of the data you used for validation. The following section contains an interpretation and suggests possible resolutions.

Case 1	Case 2	Case 3	Case 4
Dice: 0.00	Dice: nan	Dice: 0.00	Dice: 0.00
Precision: 0.00%	Precision: nan	Precision: nan	Precision: 0.00%
Recall: 0.00%	Recall: nan	Recall: 0.00%	Recall: nan

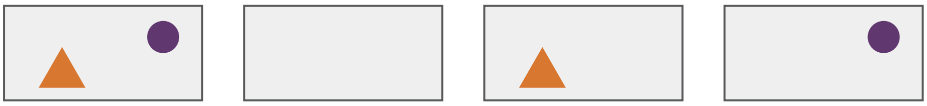

Legend

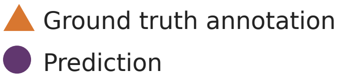

1. An image or ROI **has ground truth annotations** and **there is a prediction without any overlap**.
Dice Coefficient is 0.00 (no overlap), precision is 0.00% (all false-positives), recall is 0.00% (all false-negatives). This is a valid case, where the model is unable to segment the correct area.
Suggestion: You can check if all regions/objects were labeled correctly, because mislabeling will confuse the model.

2. An image or ROI **has no ground truth annotation**, and there is **no prediction**.
Dice Coefficient, precision and recall cannot be computed ("nan"). This is a valid case, where the model proves that it does not recognize anything when in fact there is no object.

3. An image or ROI **has ground truth annotations**, but there is **no prediction**.
Dice Coefficient is 0.00 (no overlap), precision is "nan" (false-positives cannot be computed), recall is 0.00% (all false-negatives). This usually points towards a model that is not yet able to recognize the desired label sufficiently.
Suggestion: Add more annotated regions with the desired label to your training set to improve the performance.

4. An image or ROI **has no ground truth annotation**, but **there is a prediction**.
Dice Coefficient is 0.00 (no overlap), precision is 0.00%, recall is "nan" (false-negatives cannot be computed).
Suggestion: In this case you should verify that there are really no objects or areas with the desired labels present in the image or ROI and that you did not miss annotations on actual objects.

6. Contact and Support

If you have any questions regarding your training, please send us an email at **support@ikosa.ai**.
Please copy and add your training ID `af4b5597-cc74-49f6-b0c4-c04452b87e06` in your email.

We wish you successful applications! Your IKOSA AI team.

KML VISION

KML Vision GmbH
Nikolaiplatz 4/II
8020 Graz, Austria

Mail: **support@ikosa.ai**
Phone: +43 680 1567596
